# Reactions of Trimethylaluminium: Modelling the Chemical Degradation of Synthetic Lubricants

**DOI:** 10.1002/chem.201604553

**Published:** 2016-12-05

**Authors:** Jonathan Slaughter, Andrew J. Peel, Andrew E. H. Wheatley

**Affiliations:** ^1^ Department of Chemistry University of Cambridge Lensfield Road Cambridge CB2 1EW UK), Fax

**Keywords:** esters, NMR spectroscopy, nucleophilic addition, solid-state structures, trimethylaluminium

## Abstract

In investigating and seeking to mimic the reactivity of trimethylaluminium (TMA) with synthetic, ester‐based lubricating oils, the reaction of methyl propionate **1** was explored with 1, 2 and 3 equivalents of the organoaluminium reagent. Spectroscopic analysis points to the formation of the adduct **1**(TMA) accompanied only by the low level 1:1 production of Me_2_AlOCEtMe_2_ **2** and Me_2_AlOMe **3** when an equimolar amount of TMA is applied. The deployment of excess TMA favours reaction to give **2** and **3** over **1**(TMA) adduct formation and spectroscopy reveals that in hydrocarbon solution substitution product **2** traps unreacted TMA to yield **2**(TMA). The ^1^H NMR spectroscopic observation of two Al−Me signals not attributable to free TMA and in the ratio 1:4 suggests the formation of a previously only postulated, symmetrical metallacycle in Me_4_Al_2_(μ^2^‐Me)(μ^2^‐OCEtMe_2_). In the presence of **3**, **2**(TMA) undergoes thermally induced exchange to yield Me_4_Al_2_(μ^2^‐OMe)(μ^2^‐OCEtMe_2_) **4** and TMA. The reaction of methyl phenylacetate **5** with TMA allows isolation of the crystalline product Me_2_AlOCBnMe_2_(TMA) **6**(TMA), which allows the first observation of the Me_4_Al_2_(μ^2^‐Me)(μ^2^‐OR) motif in the solid state. Distances of 2.133(3) Å (Al−Me_bridging_) and 1.951 Å (mean Al−Me_terminal_) are recorded. The abstraction of TMA from **6**(TMA) by the introduction of Et_2_O has yielded **6**, which exists as a dimer.

## Introduction

Ideas on the atmospheric reaction of chlorofluorocarbons (CFCs) have existed for more than 40 years^1^ and are well documented.^2^ Although legislation has been implemented aimed at eliminating their use,^3^ the effects of substitute refrigerants such as perfluorocarbons (PFCs) and hydrofluorocarbons (HFCs) have been the subject of subsequent scrutiny^4^ and regulation.^5^ More specifically, with emissions from (automobile) air conditioning units representing a growing climate control concern^6^ action has been initiated^7^ to avoid the use of refrigerants with a global warming potential >150 (GWP=100 year warming potential of one kg of a gas relative to one kg CO_2_).^8^ This has had the effect of phasing out greenhouse gases such as R‐134a (1,1,1,2‐tetrafluoroethane, GWP=>1000).^8^ However, illicit HFC use remains a problem, with R‐40 (chloromethane; GWP=13) having been used as a counterfeit refrigerant.^9^ This raises issues of reactivity with aluminium components in refrigeration units. Although the reaction of alkyl chlorides with aluminium under the influence of an aluminium halide catalyst is well established,^10^ it is known that reaction also proceeds in the absence of catalyst.10b, 11 In this vein, in our hands the autocatalytic formation of trialkylaluminium and (catalyst) AlCl_3_ from an alkyl chloride–aluminium mixture has been initiated by heat only.^12^ The products of reaction between R‐40 itself and aluminium include trimethylaluminium (TMA), which is potentially reactive with respect to other chemicals present. These include proprietary compound oils (e.g. RL 32H)^13^ formulated for use in conjunction with HFC refrigerants. They comprise synthetic polyolesters (POEs) which, in RL 32H itself, have a pentaerythritol core.^14^

Although the interaction of organoaluminium compounds with esters has been studied^15^ the specifics of the mechanism remain surprisingly obscure and, in particular, reaction intermediates are incompletely understood. AlEt_3_ has been reacted with esters in an equimolar ratio to give ’ate complexes that rearrange to ketones and aldehydes.^16^ Studies using TMA have explored the formation of donor–acceptor complexes and their derivatization with excess aluminium reagent.^17^ Moreover, the deployment of excess TMA at high temperature has incurred double methylation and tertiary alcohol formation. However, mechanistic insights were limited to the alternative use of Me_2_AlCl.^18^ Although the formation of hemialkoxides has been postulated based on the derivatization of ketones and aldehydes using alkylaluminiums,^19^ these species have not hitherto been recorded in ester‐based systems. Meanwhile, the use of excess TMA has been reported in the alkylation of acetates,^20^ and the ketonization of heteroaromatic esters using 1 equivalent of TMA has been reported.^21^, ^22^ Reaction selectivity has been investigated, with TMA used at low temperature^23^ in the stereoselective reduction of cyclic ketones^24^ to give neoliacinic acid.^25^ The reaction was done in the presence of ancillary ester groups, with competing transesterification proving controllable.^26^

The expected by‐product of ester reaction with TMA, Me_2_AlOR, has been the subject of extensive study.^27^ However, this has tended to focus not upon its synthesis as a by‐product of ketonization reactions but rather on the oxophilic derivatization of Al−C bonds^28^ by moisture^29^ or oxygen.^30^ From a structural point of view, aluminium organooxide formation^31^ and di‐/trimerization is well established,^32^ for example, the simple aluminium alkoxide Me_2_AlOMe has been shown to be trimeric.^33^, ^34^

In this work we model the reaction of TMA with synthetic POEs and elucidate intermediates along the reaction pathway between TMA and esters in general for the first time. Structure and stability are monitored for intermediate complexes and solution data clarify the reaction stoichiometry.

## Results and Discussion

**Figure 1:**
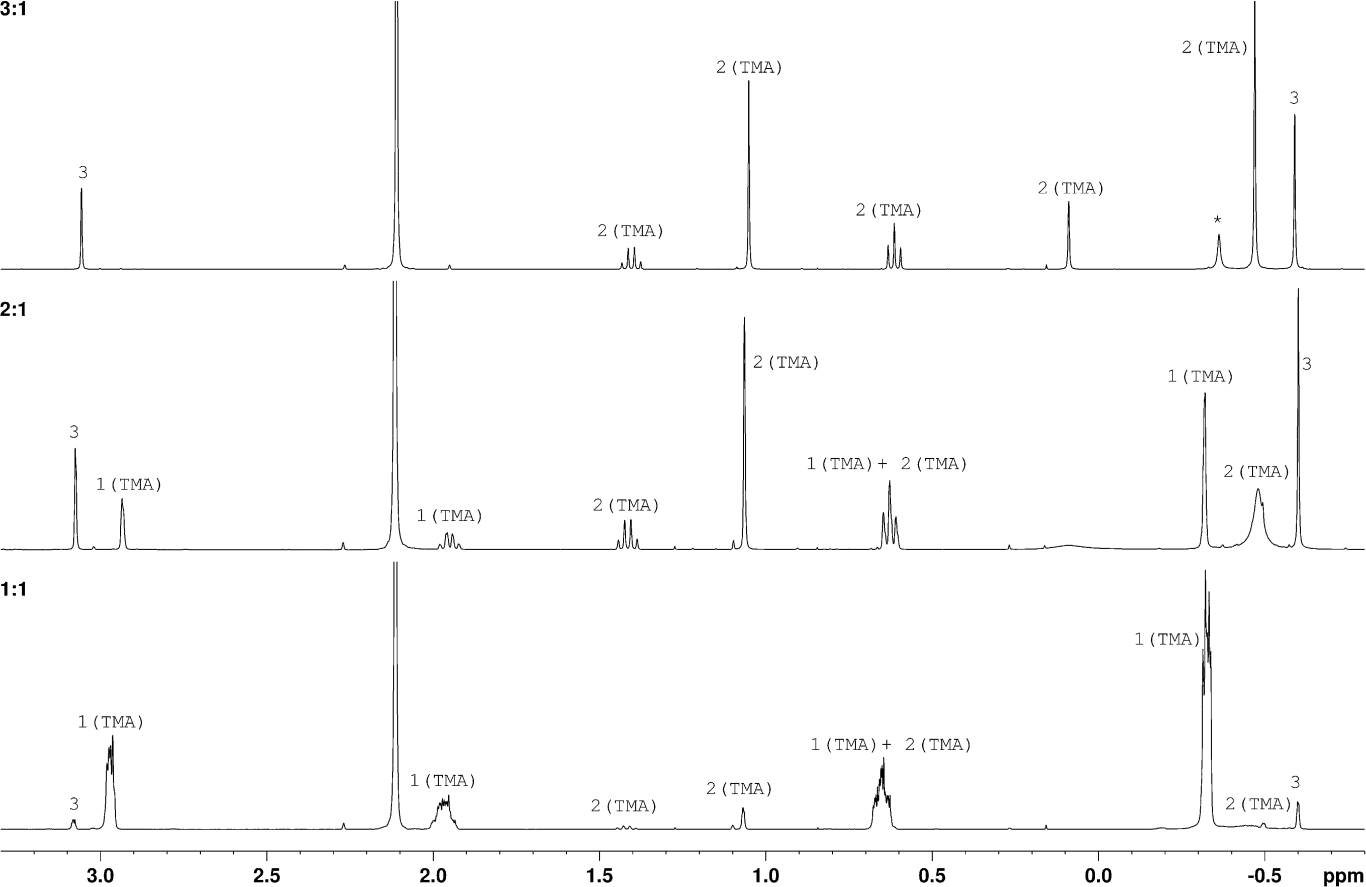
The ^1^H spectra of aliquots from the reaction between TMA and methyl propionate **1** in toluene (*δ*_H_=2.11 ppm) employing 3:1 (top), 2:1 (middle) and 1:1 (bottom) stoichiometries. The solvent is [D_6_]benzene. *Free TMA.

**Scheme 1:**
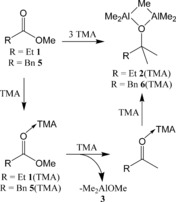
The conversion of **1**/**5** into **2**(TMA)/**6**(TMA).

The ability of alkylaluminium compounds to be autocatalytically generated through the action of alkyl chlorides on aluminium metal has led us to seek to model the potential reactivity of lubricant oils used in industrial refrigeration units with respect to TMA. Reactions involving a simple aliphatic ester were undertaken whereby TMA in toluene was initially added dropwise to methyl propionate **1** (1:1) under a N_2_ atmosphere at −78 °C. Though this system failed to readily produce isolable products, the observation of a pale‐green colour upon heating, which disappeared when left to cool to room temperature, suggested the interaction of ester and TMA and led to further investigations. Accordingly, an excess of TMA (see the Supporting Information, Figure S1) was added to **1** (3:1 TMA:**1**) under N_2_ at −78 °C. After reaching room temperature the solution was stirred for 2 hours, whereupon the NMR spectra of an aliquot were collected. ^1^H NMR spectroscopy and COSY suggested the formation of two species (Figure 1, top), with ^13^C NMR spectroscopy confirming the complete absence not only of ester but of C=O groups from each species (see Figure S3 in the Supporting Information). These data suggest that 2:1 reaction of TMA with ester has occurred, one equivalent of TMA expelling methoxide to induce the formation of a reactive EtMeC=O intermediate alongside Me_2_AlOMe **3** (*δ*_H_=3.06, −0.59 ppm)^32^ before a second equivalent of TMA has reacted with the ketone to give the dimethylaluminium alkoxide Me_2_AlOCEtMe_2_ **2**. Integrals of peaks at *δ*_H_=0.61 (**2**) and 3.06 ppm (**3**) suggest the two products to be present in a 1:1 ratio. Lastly, the observation that signals at *δ*_H_=−0.47 and −0.59 ppm reveal relative integrals of 2:1 leads us to speculate that **2** traps the final (unreacted) equivalent of TMA present to give Me_2_AlOCEtMe_2_(TMA) **2**(TMA) (Scheme 1). Based on these spectroscopic data we attribute what would be an unusual 4‐membered Al_2_OC metallacycle to this adduct, in which four Me groups are equivalent, with the terminal groups (Me_t_) resonating at *δ*_H_=−0.47 ppm while the unique bridging group (Me_b_) resonates at *δ*_H_=0.09 ppm. This view is reinforced by ^13^C NMR spectroscopy (Supporting Information, Figure S3), which reveals a sharp signal at *δ*_C_=−4.1 ppm due to the bridging methyl in **2**(TMA) and broad signals for terminal AlMe groups at *δ*_C_=−6.7 and −10.7 ppm in **2**(TMA) and **3**, respectively. Lastly, it is consistent with ^27^Al NMR spectroscopic evidence, which reveals a broad signal at *δ*_Al_=156.2 ppm (Supporting Information, Figure S3) attributable to 4‐coordinate aluminium.^35^

To further clarify the co‐formation of putative **2**(TMA) and **3**, the same synthetic process was repeated using 2:1 and 1:1 TMA:**1** ratios (Figure 1, middle and bottom). The ^1^H NMR spectrum of the last of these systems is dominated by the formation of a complex between **1** and TMA (Figure 1, bottom), with signals from unreacted **1** at *δ*_H_ *=*3.32 (s), 1.99 (q) and 0.93 (t) ppm (c.f. Supporting Information Figure S2) moved to *δ*_H_ *=*2.98 (m), 1.97 (m) and 0.65 (m) ppm, whereas coordinated TMA is revealed downfield (*δ*_H_ *=*−0.33 ppm) of free TMA (*δ*_H_ *=*−0.35 ppm, Figure S1). ^13^C NMR spectroscopy reveals retention of a modified ester in **1**(TMA) (*δ*_C_ *=*181.4 ppm, Figure S4; c.f. *δ*_C_ *=*173.9 ppm in **1**, Figure S2 in the Supporting Information) and the presence of coordinated TMA (*δ*_C_ *=*−7.7 ppm). However, consistent with previous reports,^20^, ^26^ negligible conversion of the complex **1**(TMA) into addition product is observed in this system. In contrast, the 2:1 TMA:**1** system reveals not only the interaction of **1** with TMA to yield adduct **1**(TMA) but also the development of two further species (Figure 1, middle). Hence, signals attributable to **1**(TMA) are noted in the ^1^H NMR spectrum at *δ*_H_ *=*2.94, 1.95, 0.63 and −0.32 ppm and ^13^C NMR spectroscopy reveals retention of the ester function at *δ*_C_ *=*181.8 ppm (Supporting Information, Figure S5). However, this adduct is now less populous in solution than two other distinct species. One of these presents signals at *δ*_H_ *=*3.08 and −0.60 ppm and is attributable to evolving Me_2_AlOMe **3**, whereas the other is consistent with the alkoxide **2**. The high field region of the spectrum demonstrates ^1^H NMR resonances at *δ*_H_ *=*0.09 and −0.49 ppm (3 H and 12 H, respectively), neither of which correspond to unreacted TMA. This reinforced the view already expressed (Figure 1, top) that an adduct, **2**(TMA), exists in solution, that high field signals are attributable to one bridging (AlMe_b_) and four terminal (AlMe_t_) AlMe groups, respectively, in a Me_2_Al(μ^2^‐Me)AlMe_2_ fragment and that **2**(TMA) is symmetrical, metallacyclic Me_4_Al_2_(μ^2^‐Me)(μ^2^‐OCEtMe_2_) (Scheme 1). In a similar vein, ^13^C NMR spectroscopy showed resonances at *δ*_C_ *=*−4.6 and −7.7 ppm due to AlMe_b_ and AlMe_t_, respectively, the latter representing the superposition of signals attributable to both **1**(TMA) and **2**(TMA). Meanwhile, **3** was now clearly shown by the presence of a signal at *δ*_C_ *=*−11.1 ppm. Both **2**(TMA) and **3** were retained in the 3:1 system, with **1**(TMA) now completely absent and a small amount of unreacted TMA identified at *δ*_C_ *=*−0.36 ppm (Figure 1, top and Supporting Information Figure S3). ^27^Al NMR spectroscopy evidenced the trend from **1**(TMA) towards the formation of **2**(TMA) and **3** by the gradual replacement of a dominant signal at *δ*_Al_ *=*185.0 ppm in the 1:1 system (carbonyl‐bonded 4‐coordinate Al) with a signal at *δ*_Al_ *=*156.2 ppm in the 3:1 system (alkoxide‐bonded 4‐coordinate Al; Supporting Information Figures S3–S5).

**Figure 2:**
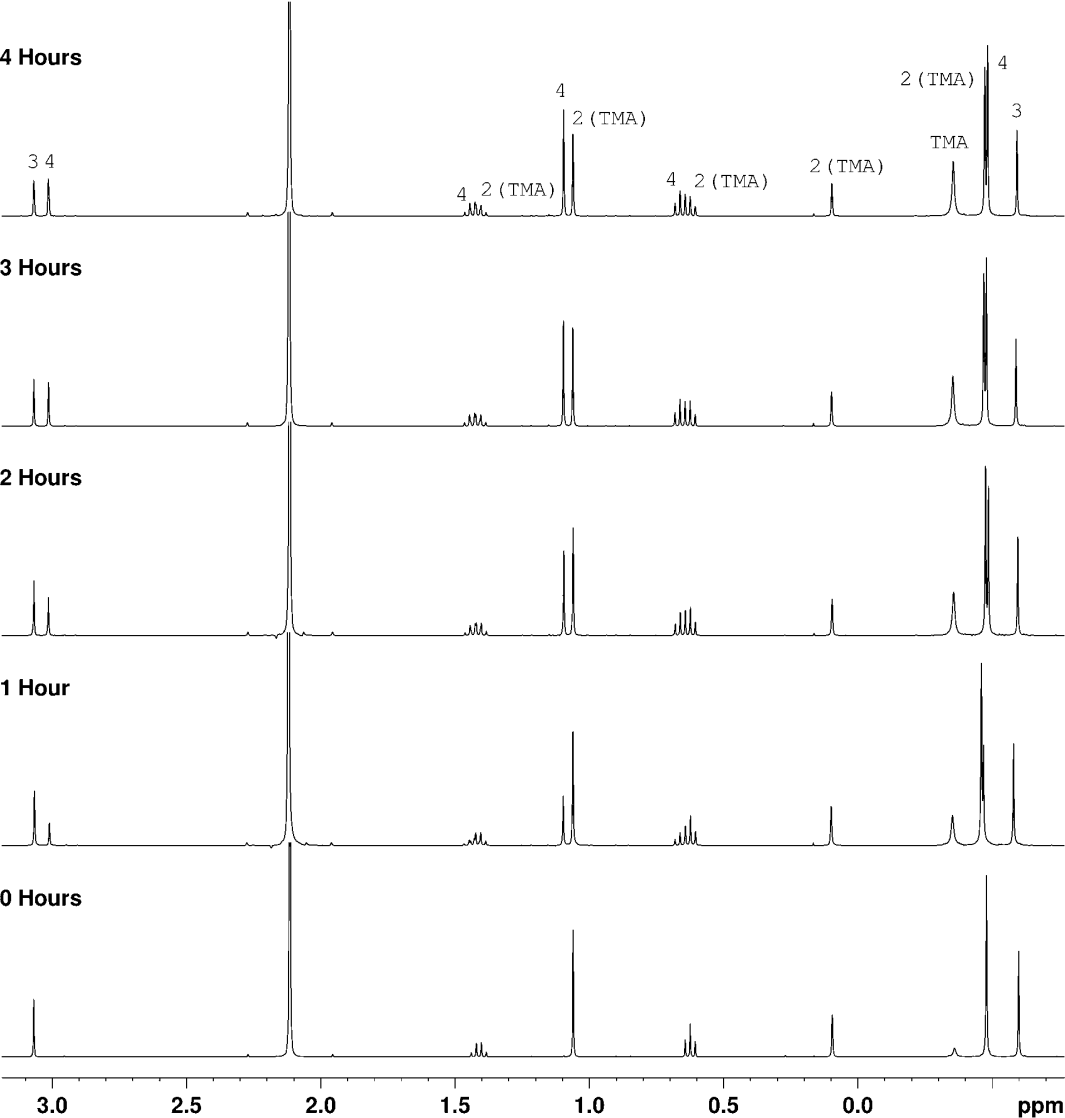
The ^1^H spectra (locked using [D_6_]benzene) of aliquots from the thermally induced reaction between **2**(TMA) and 1 equiv. of **3** in toluene (*δ*_H_=2.11 ppm) after time (*t*)=0–4 hours.

**Scheme 2:**
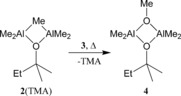
The thermally induced conversion of **2**(TMA) into **4**.

Spectroscopy points to an Me_4_Al_2_(μ^2^‐Me)(μ^2^‐OCEtMe_2_) formulation based on a symmetrical OAl_2_C metallacycle for **2**(TMA). However, although this is similar to motifs previously proposed,^19^ the thermal stability of such a motif has not hitherto been reported. With this in mind, the reaction mixture resulting from the introduction of TMA in toluene to **1** in a 3:1 ratio (spectroscopically characterized as ostensibly a 1:1 mixture of **2**(TMA) and **3**, Figure 1, top) was heated to reflux for 4 hours. NMR spectroscopic analysis of aliquots obtained after *t=*0, 1, 2, 3 and 4 hours revealed a gradual thermal rearrangement (Figure 2 and Supporting Information Figure S6), with the spectra demonstrating the in situ reformation of free TMA (*δ*_H_ *=*−0.36 ppm (^1^H after 4 hours)) alongside that of the new complex **2**(**3**)=Me_4_Al_2_(μ^2^‐OMe)(μ^2^‐OCEtMe_2_) **4** (Scheme 2). Evidence for the symmetry of an O_2_Al_2_ ring in **4** comes from the development of a single Me_t_ signal at *δ*_H_ *=*−0.48 ppm. Meanwhile, residual **2**(TMA) (*δ*_H_ *=*−0.47 ppm) and **3** (*δ*_H_ *=*−0.59 ppm) remain clearly identifiable. Integrals suggest that thermal rearrangement of **2**(TMA)+**3** to give **4**+TMA proceeds to around 54 % completion after 4 hours under the conditions used.

Further investigation of the thermal rearrangement of **2**(TMA) focused on the reaction mixtures resulting from the 1:1 and 2:1 reaction of TMA with **1** (spectroscopically characterized as ostensibly **1**(TMA) (Figure 1, bottom) and a mixture of **1**(TMA), **2**(TMA) and **3** (Figure 1, middle), respectively) being heated to reflux for 2 hours. Data elucidate the processes in Equations 1 and 2;(1), (2), (3)

(1) 1(TMA)+2TMA→2(TMA)+3

(2) 2(TMA)+3⇌4+TMA

(3) 4⇌2+3

**Figure 3:**
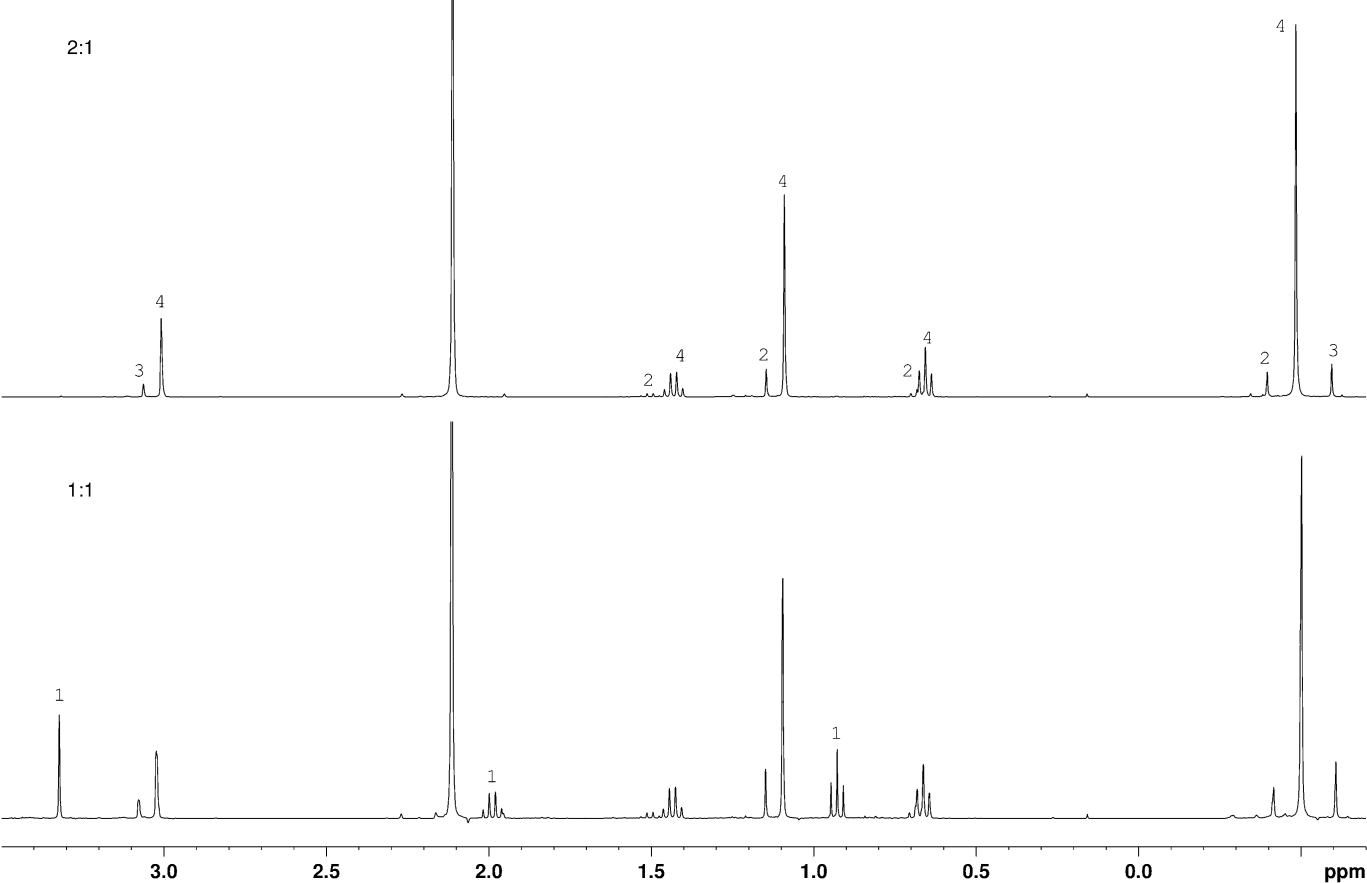
The ^1^H spectra (locked using [D_6_]benzene) of aliquots from the thermally induced reaction of mixtures resulting from the 2:1 (top) and 1:1 (bottom) TMA:**1** systems obtained after 2 hours. Toluene is seen at *δ*_H_=2.11 ppm.

The use of 1:1 **1**:TMA without heating results in very limited reaction, with only traces of **2**(TMA) and **3** existing alongside (dominant) **1**(TMA) (*δ*_H_ *=*2.98 ppm, Figure 1, bottom). Even heating fails to completely consume **1** and instead around 50 % unreacted **1** can clearly be seen after 2 hours (*δ*_H_ *=*3.32 ppm, Figure 3, bottom). This is explained by viewing **1** as reacting with 3 equivalents of TMA to yield **2**(TMA) and **3**, which then undergoes thermal exchange to give **4**+TMA. This latterly generated TMA can then react with remaining **1**, eventually converting half the available **1** into **4**. In the 1:2 **1**:TMA system, the greater amount of TMA present aids the formation of **2**(TMA)+**3** [Eq. (1)] and Figure 3, top). These then act as a source of further **4**+TMA [Eq. (2)]. The eventual consequence of this cycle is the complete removal of both **1**(TMA) and TMA from the system. This explains the formation of **4** only in this system. However, remnant **3** is also observed. This can be understood by the appearance of a further species (*δ*_H_ *=*1.50, 1.15, 0.68, −0.40 ppm), which we attribute to **2**. (We speculate that this product forms a dimer or trimer in solution based on the observation of a ^27^Al NMR signal that, at *δ*_Al_ *=*155.1 ppm, is consistent with tetracoordinate metal.) It appears then that **4** is in equilibrium with it constituents [Eq. (3)]; a fact most clearly suggested by noting the 1:1 ratio of the signals at *δ*_H_ *=*−0.40 and −0.59 ppm (**2** and **3**, respectively). Based on ^1^H NMR spectroscopy, this equilibrium lies heavily on the side of **4** (ca. 88:12 **4**:(**2**+**3**)).

**Figure 4:**
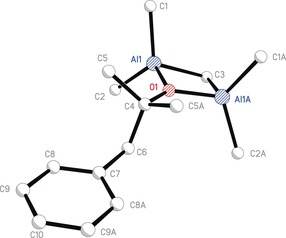
Molecular structure of **6**(TMA), H‐atoms omitted. Selected bond lengths (Å) and angles (°): O1−Al1 1.8240(15), C1−Al1 1.952(3), C2−Al1 1.949(3), C3−Al1 2.133(3), C4−O1 1.468(4), Al1‐C3‐Al1A 79.50(12), Al1‐O1‐Al1A 96.79(10), C3‐Al1‐O1 90.97(8), C1‐Al1‐C2 120.17(12).

**Figure 5:**
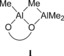
The previously documented bis(oxyphenyl) structure type.

Repeated attempts to isolate crystalline products of reaction between methyl propionate **1** and TMA proved fruitless on account of a low melting point and led to the replacement of **1** with methyl phenylacetate **5** (Supporting Information, Figure S7) in an attempt to crystallographically verify the identities/structures of ester decomposition products. Hence, TMA in toluene was added dropwise to **5** (1:1, 2:1 or 3:1 TMA:**5**). NMR spectroscopic analysis of the resulting mixture revealed similar behaviour to that noted for the methyl propionate system, with the formation of initial adduct **5**(TMA) in the presence of 1 equivalent of TMA followed by reaction to give **6**(TMA) and **3** in the presence of more than 1 equivalent of TMA (Scheme 1 and Supporting Information Figures S8–S10). As with **2**(TMA), the capture of excess TMA by **6** could be inferred from the ^1^H NMR spectroscopic observation of Al‐bonded Me groups at high field (*δ*_H_ *=*−0.42 ppm (Me_t_) and *δ*_H_ *=*0.13 ppm (Me_b_)) in a 4:1 ratio alongside retention of the singlet at *δ*_H_ *=*−0.59 ppm due to **3** (see above). ^13^C NMR spectroscopy reinforced the co‐presence of **3** alongside **6**(TMA) through the observation of a broad high field resonance at *δ*_C_ *=*−11.1 ppm (**3**) alongside signals at *δ*_C_ *=*−4.5 (**6**(TMA), Me_b_) and −7.0 ppm (**6**(TMA), Me_t_). For the 3:1 TMA:**5** combination, the liquid remaining after reaction was reduced in volume and stored at 4 °C for 1 day to produce colourless crystals that analyzed as a mixture of Me_2_AlOCBnMe_2_(TMA) (**6**(TMA); Bn=CH_2_Ph) and **3**. It was now possible to confirm the identity of **6**(TMA) as Me_4_Al_2_(μ^2^‐Me)(μ^2^‐OCBnMe_2_), with X‐ray diffraction establishing the symmetry of the Al_2_OC ring formed by the capture of TMA and the presence of the expected terminal (Me_t_) and μ^2^‐bridging (Me_b_) methyl groups (Figure 4). The result is the observation of two distinct classes of Al−Me interaction; Al−Me_b_ 2.133(3) Å, Al−Me_t_ 1.951 Å (mean). The only previous report of which we are aware of diffraction data for the symmetrical metallacyclic motif reported herein lies with the electron diffraction analysis of the hemialkoxide Me_2_AlO*t*Bu(TMA) in the gas phase (Al−Me_b_ 2.103(10) Å, Al−Me_t_ 1.948(7) Å (mean)).^36^, ^37^ In the solid state the Al_2_OC motif has only very rarely been recorded, with a search of the Cambridge Crystallographic Database returning just seven results. Of these, only five show trapped TMA, demonstrating the highly unusual nature of this phenomenon. The nearest analogues of **6**(TMA) are based on asymmetric bis(oxyphenyl) structures of type **I** (Figure 5) demonstrated by tetraaluminium bis(bis(oxyphenyl)methyl)anthracene and ‐dibenzofuran complexes^38^ and the bisaluminium derivative of a 1,1′‐bis‐2,2′‐oxynaphthyl ligand.^39^ A type **I** motif has been recorded once also in heterobimetallic Al–Ti chemistry.^40^

**Figure 6:**
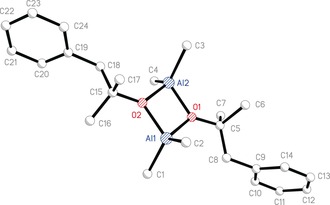
Molecular structure of **6**_2_, H‐atoms and minor disorder omitted. Selected bond lengths (Å) and angles (°): O1−Al1 1.8457(10), O1−Al2 1.8493(10), O2−Al1 1.8526(10), O2−Al2 1.8447(10), C1−Al1 1.9522(16), C2−Al1 1.9559(16), C3−Al2 1.9565(15), C4−Al2 1.9492(15), C5−O1 1.434(10), C15−O2 1.4615(16), Al1‐O1‐Al2 98.76(5), Al1‐O2‐Al2 98.68(5), O1‐Al1‐O2 81.20(4), O1‐Al2‐O2 81.32(4).

The repeated observation that both **2**(TMA) and **6**(TMA) form alongside Me_2_AlOMe **3** and that crystalline **6**(TMA) is isolated contaminated by **3** led to attempts to separate the components. Efforts here took two forms. In one set of experiments, the solvent mixture was modified post‐synthetically. Hence, the recrystallization of **6**(TMA) (leading to the crystal structure shown in Figure 4) gave a crystalline material that analyzed by ^1^H NMR spectroscopy as an approximately 1:1 mixture of **6**(TMA) and **3** (see Experimental Section, co‐synthesis of **6**(TMA) and **3**, Method 1). This ratio accurately reflected that of the two products generated in the reaction, which point was simply evidenced by analysing an aliquot of the reaction mixture (see Experimental Section, spectroscopic characterization of **5**+3 TMA). In contrast, the introduction of hexane prior to recrystallization vastly improved the purity with which crystalline **6**(TMA) could be isolated (10:1 **6**(TMA):**3** by ^1^H NMR spectroscopy; see Experimental Section, co‐synthesis of **6**(TMA) and **3**, Method 2). An alternative approach involved attempting to solvate one component of the **3**/**6**(TMA) mixture using a Lewis base. With this in mind, TMA in toluene was added to methyl phenylacetate in a 3:1 ratio under N_2_ at −78 °C. Removal of toluene was followed by the addition of excess Et_2_O. This resulted in the precipitation of a white solid, which was recrystallised by heating to give a solution and then storing at room temperature to produce colourless blocks. ^1^H NMR spectroscopic analysis suggested the presence of Ph but not of Et_2_O and high field signals previously attributed to TMA were absent. These data suggest the abstraction of TMA as an ether solvate,^41^ leading to the crystallization of **6**. This was confirmed crystallographically by the observation of a simple dimer based on an (AlO)_2_ core of a type common in aluminium organooxide chemistry (Figure 6).27b, 28a

## Conclusion

In summary, the autocatalytic nature of the reaction between alkylchlorides and aluminium to give AlCl_3_ and TMA has led us to study the reactions of TMA with model esters that mimic synthetic lubricants of the type used in industrial refrigeration units. Reaction has been found to be heavily dependent on stoichiometry. Hence, the treatment of either methyl propionate or methyl phenylacetate with 1 equivalent of TMA gave predominantly the corresponding ester‐TMA adducts **1**(TMA) or **5**(TMA) with only nominal reaction occurring to give a 1:1 mixture of Me_4_Al_2_(μ^2^‐Me)(μ^2^‐OCRMe_2_) (R=Et **2**(TMA) or Bn **6**(TMA)) and Me_2_AlOMe **3**. In either case reaction was encouraged by adding more TMA, with full conversion occurring for a 3:1 TMA:ester ratio. Spectroscopy clarified the trapping of **2** and **6** by TMA, suggesting the structures of the resulting adducts to be based on symmetrical OAl_2_C metallacycles. For **2**(TMA), the presence of concurrently formed **3** induced thermal exchange to yield a more stable metallacycle in Me_4_Al_2_(μ^2^‐OMe)(μ^2^‐OCEtMe_2_) **4**. The ability to isolate **6**(TMA) from a mixture of reaction products proved highly solvent dependent, with by‐product Me_2_AlOMe **3** largely retained in hexane solution whereas **6**(TMA) crystallized, allowing confirmation of the rare OAl_2_C heterocycle. Efforts are now underway to extend this study to the use of more complex diesters and pentaerythritol‐based esters, the latter representing a close analogue of bona fide POEs.

## Experimental Section

### General synthetic and analytical details

Reactions and manipulations were carried out under dry nitrogen, using double manifold and glove‐box methods. Solvents were distilled off sodium‐potassium amalgam (Et_2_O, hexane) immediately before use. Methyl propionate (99 %) and methyl phenylacetate (>99 %) were purchased from Sigma–Aldrich and stored over molecular sieve (4 Å). TMA (2.0 m in toluene) was purchased from Sigma–Aldrich and used as received. Elemental analysis was carried out on a PerkinElmer 240 Elemental Analyser. NMR data were collected on a Bruker Avance III HD 400 MHz Smart Probe FT NMR spectrometer (400.130 MHz for ^1^H, 100.613 MHz for ^13^C, 104.261 for ^27^Al). Spectra were obtained at 25 °C. For ^1^H and ^13^C, chemical shifts are internally referenced to deuterated solvent and calculated relative to TMS. For ^27^Al, an external reference was used (1 m AlCl_3_(H_2_O)_6_ in D_2_O). Chemical shifts are expressed in *δ* ppm. The following abbreviations are used: br=broad, m=multiplet, q=quartet, s=singlet, sh=shoulder, t=triplet.

### Crystallographic details

Crystals were transferred from the mother liquor to a drop of perfluoropolyether oil mounted upon a microscope slide under cold nitrogen gas.^42^ Suitable crystals were attached to the goniometer head via a MicroLoop^TM^, which was then centred on the diffractometer. Data were collected on a Bruker D8 Quest (Cu‐Kα, *λ*=1.54184 Å), equipped with an Oxford Cryosystems low‐temperature device. Structures were solved using SHELXT, with refinement, based on *F* ^2^, by full‐matrix least squares.^43^ Non‐hydrogen atoms were refined anisotropically and a riding model with idealized geometry was employed for the refinement of H‐atoms. For **6**_2_ one BnMe_2_C group was modelled as disordered, though separate positions for the phenyl group could not be refined satisfactorily. The occupancy was refined, with restraints placed upon both the 1,2‐ and 1,3‐distances and upon the anisotropic atomic displacement parameters. CCDC 1504652 and 1504653 contain the supplementary crystallographic data for this paper. These data are provided free of charge by The Cambridge Crystallographic Data Centre.

### Spectroscopic characterization of EtC(O)OMe 1+TMA reaction mixtures

TMA (1.5, 3.0 or 4.5 mL, 3, 6 or 9 mmol, 2.0 m in toluene) was added dropwise to methyl propionate **1** (0.29 mL, 3 mmol) under a N_2_ atmosphere at −78 °C before being allowed to reach room temperature. The resulting solution was stirred for 2 hours at this temperature. An aliquot (0.1 mL) was mixed with [D_6_]benzene (0.7 mL) and analyzed by NMR spectroscopy, revealing **1**(TMA), **2**(TMA) and **3**.

1:1 **1**:TMA. ^1^H NMR spectroscopy (400 MHz, [D_6_]benzene): *δ*=3.08 (s, 0.2 H; **3** OMe), 2.98 (m, 3 H; **1**(TMA), OMe), 1.97 (m, 2 H; **1**(TMA), CH_2_), 1.42 (q, 0.1 H; **2**(TMA) CH_2_), 1.07 (s, 0.3 H; **2**(TMA) Me), 0.65 (m, 3.2 H; **1**(TMA)+**2**(TMA) CH_2_ *Me*), 0.16 (s, 0.1 H; **2**(TMA) Me_b_), −0.33 (m, 9 H; **1**(TMA), AlMe), −0.49 (s, 0.3 H; **2**(TMA) Me_t_), −0.60 ppm (s, 0.4 H; **3** Me); ^13^C NMR (100 MHz, [D_6_]benzene): *δ*=181.4 (**1**(TMA) CO), 79.5 (**2**(TMA) CO), 53.8 (**1**(TMA) OMe), 50.4 (**3** OMe), 36.6 (**2**(TMA) CH_2_), 27.7 (**1**(TMA) *CH_2_*Me+**2**(TMA) Me), 8.8 (**2**(TMA) CH_2_ *Me*), 8.2 (**1**(TMA) CH_2_ *Me*), −7.7 ppm (**1**(TMA) AlMe); ^27^Al NMR (104 MHz, [D_6_]benzene): *δ*=185.0 (**1**(TMA)), 157.7 ppm (**2**(TMA)+**3**).

1:2 **1**:TMA. ^1^H NMR spectroscopy (400 MHz, [D_6_]benzene): *δ*=3.08 (s, 3 H; **3** OMe), 2.94 (s, 2.4 H; **1**(TMA), OMe), 1.95 (q, 1.6 H; **1**(TMA), CH_2_), 1.42 (q, 2 H; **2**(TMA) CH_2_), 1.07 (s, 6 H; **2**(TMA) Me), 0.63 (t, 5.4 H; **1**(TMA)+**2**(TMA) CH_2_ *Me*), 0.09 (br, s, 3 H; **2**(TMA) Me_b_), −0.32 (s, 7.1 H; **1**(TMA), AlMe), −0.49 (s, br, 12 H; **2**(TMA) Me_t_), −0.60 ppm (s, 6 H, **3** Me); ^13^C NMR (100 MHz, [D_6_]benzene): *δ*=181.8 (**1**(TMA) CO), 79.5 (**2**(TMA) CO), 54.0 (**1**(TMA) OMe), 50.4 (**3** OMe), 36.6 (**2**(TMA) CH_2_), 27.7 (**1**(TMA) *CH_2_*Me+**2**(TMA) Me), 8.8 (**2**(TMA) CH_2_ *Me*), 8.2 (**1**(TMA) CH_2_ *Me*), −4.6 (**2**(TMA) Me_b_), −7.7 (br, **1**(TMA)+**2**(TMA) Me_t_), −11.1 ppm (**3** Me); ^27^Al NMR (104 MHz, [D_6_]benzene): *δ*=179.7 (sh., **1**(TMA)),153.9 ppm (**2**(TMA)+**3**).

1:3 **1**:TMA. ^1^H NMR spectroscopy (400 MHz, [D_6_]benzene): *δ*=3.06 (s, 3 H; **3** OMe), 1.41 (q, 2 H; **2**(TMA) CH_2_), 1.05 (s, 6 H; **2**(TMA) Me), 0.61 (t, 3 H; **2**(TMA) CH_2_ *Me*), 0.09 (s, 3 H; **2**(TMA) Me_b_), −0.36 (s, 3.5 H; TMA), −0.47 (s, 12 H; **2**(TMA) Me_t_), −0.59 ppm (s, 6 H; **3** Me); ^13^C NMR (100 MHz, [D_6_]benzene): *δ*=79.9 (**2**(TMA) CO), 50.8 (**3** OMe), 37.0 (**2**(TMA) CH_2_), 28.1 (**2**(TMA) Me), 9.2 (**2**(TMA) CH_2_ *Me*), −4.1 (**2**(TMA) Me_b_), −6.7 (br, **2**(TMA) Me_t_), −10.7 ppm (br, **3** Me); ^27^Al NMR (104 MHz, [D_6_]benzene): *δ*=156.2 ppm (**2**(TMA)+**3**).

### Thermal stability of 1:3 EtC(O)OMe 1:TMA reaction mixture

The reaction mixture at the end of the 1:3 reaction of **1** with TMA in toluene (to give **2**(TMA) and **3**; see above) was heated to reflux for 4 hours using an oil bath to form Me_4_Al_2_(μ^2^‐OMe)(μ^2^‐OCEtMe_2_) **4** and TMA. Aliquots (0.1 mL) were diluted with [D_6_]benzene (0.7 mL) and analyzed by NMR spectroscopy after time, *t*,=0 (before heating), 1, 2, 3 and 4 hours.

*t=*0 hr. ^1^H NMR spectroscopy (400 MHz, [D_6_]benzene): *δ*=3.07 (s, 3 H; **3** OMe), 1.41 (q, 2 H; **2**(TMA) CH_2_), 1.06 (s, 6 H; **2**(TMA) Me), 0.62 (t, 3 H; **2**(TMA) CH_2_ *Me*), 0.10 (s, 3 H; **2**(TMA) Me_b_), −0.36 (s, br, 1.5 H; TMA), −0.48 (s, 12 H; **2**(TMA) Me_t_), −0.60 ppm (s, 6 H; **3** Me); ^13^C NMR (100 MHz, [D_6_]benzene): *δ*=79.5 (**2**(TMA) CO), 50.4 (**3** OMe), 36.6 (**2**(TMA) CH_2_), 27.7 (**2**(TMA) Me), 8.8 (**2**(TMA) CH_2_ *Me*), −4.5 (**2**(TMA) Me_b_), −7.4 (br, **2**(TMA) Me_t_), −10.9 ppm (br, **3** Me).

*t=*1 hr. ^1^H NMR spectroscopy (400 MHz, [D_6_]benzene): *δ*=3.06 (s, 2 H; **3** OMe), 3.00 (s, 1 H; **4** OMe), 1.44 (q, 0.7 H; **4** CH_2_), 1.41 (q, 1.3 H; **2**(TMA) CH_2_), 1.09 (s, 2 H; **4** Me), 1.05 (s, 4 H; **2**(TMA) Me), 0.65 (t, 1 H; **4** CH_2_ *Me*), 0.61 (t, 2 H; **2**(TMA) CH_2_ *Me*), 0.09 (s, 2 H; **2**(TMA) Me_b_), −0.36 (s, br, 3 H; TMA), −0.47 (s, 8 H; **2**(TMA) Me_t_), −0.48 (s, 4 H; **4** Me_t_), −0.59 ppm (s, 4 H; **3** Me); ^13^C NMR (100 MHz, [D_6_]benzene): *δ*=79.5 (**2**(TMA) CO), 77.0 (**4** CO), 50.4 (**3** OMe), 48.4 (**4** OMe), 37.1 (**4** CH_2_), 36.6 (**2**(TMA) CH_2_), 28.1 (**4** Me), 27.7 (**2**(TMA) Me), 8.9 (**4** CH_2_ *Me*), 8.8 (**2**(TMA) CH_2_ *Me*), −4.5 (**2**(TMA) Me_b_), −7.3 (**4** Me_t_), −7.5 (**2**(TMA) Me_t_), −9.5 ppm (**3** Me).

*t=*2 hr. ^1^H NMR spectroscopy (400 MHz, [D_6_]benzene): *δ*=3.07 (s, 1.7 H; **3** OMe), 3.01 (s, 1.3 H; **4** OMe), 1.43 (q, 0.8 H; **4** CH_2_), 1.41 (q, 1.2 H; **2**(TMA) CH_2_), 1.09 (s, 2.5 H; **4** Me), 1.06 (s, 3.5 H; **2**(TMA) Me), 0.66 (t, 1.3 H; **4** CH_2_ *Me*), 0.62 (t, 1.7 H; **2**(TMA) CH_2_ *Me*), 0.10 (s, 1.5 H; **2**(TMA) Me_b_), −0.36 (s, br, 4.5 H; TMA), −0.48 (s, 7 H; **2**(TMA) Me_t_), −0.49 (s, 5 H; **4** Me_t_), −0.60 ppm (s, 3.5 H; **3** Me); ^13^C NMR (100 MHz, [D_6_]benzene): *δ*=79.5 (**2**(TMA) CO), 77.0 (**4** CO), 50.4 (**3** OMe), 48.3 (**4** OMe), 37.1 (**4** CH_2_), 36.6 (**2**(TMA) CH_2_), 28.1 (**4** Me), 27.7 (**2**(TMA) Me), 8.9 (**4** CH_2_ *Me*), 8.8 (**2**(TMA) CH_2_ *Me*), −4.5 (**2**(TMA) Me_b_), −7.4 (br, **4** Me_t_+**2**(TMA) Me_t_), −9.5 ppm (**3** Me).

*t=*3 hr. ^1^H NMR spectroscopy (400 MHz, [D_6_]benzene): *δ*=3.07 (s, 1.5 H; **3** OMe), 3.01 (s, 1.5 H; **4** OMe), 1.44 (q, 1 H; **4** CH_2_), 1.41 (q, 1 H; **2**(TMA) CH_2_), 1.10 (s, 3 H; **4** Me), 1.06 (s, 3 H; **2**(TMA) Me), 0.66 (t, 1.5 H; **4** CH_2_ *Me*), 0.62 (t, 1.5 H; **2**(TMA) CH_2_ *Me*), 0.10 (s, 1.5 H; **2**(TMA) Me_b_), −0.35 (s, br, 4.5 H; TMA), −0.47 (s, 6 H; **2**(TMA) Me_t_), −0.48 (s, 6 H; **4** Me_t_), −0.59 ppm (s, 3 H; **3** Me); ^13^C NMR (100 MHz, [D_6_]benzene): *δ*=79.5 (**2**(TMA) CO), 77.0 (**4** CO), 50.4 (**3** OMe), 48.3 (**4** OMe), 37.1 (**4** CH_2_), 36.6 (**2**(TMA) CH_2_), 28.1 (**4** Me), 27.7 (**2**(TMA) Me), 8.9 (**4** CH_2_ *Me*), 8.8 (**2**(TMA) CH_2_ *Me*), −4.5 (**2**(TMA) Me_b_), −7.5 (br, **4** Me_t_+**2**(TMA) Me_t_), −9.5 ppm (**3** Me).

*t=*4 hr. ^1^H NMR spectroscopy (400 MHz, [D_6_]benzene): *δ*=3.07 (s, 1.4 H; **3** OMe), 3.02 (s, 1.6 H; **4** OMe), 1.43 (q, 1.1 H; **4** CH_2_), 1.41 (q, 0.9 H; **2**(TMA) CH_2_), 1.10 (s, 3.3 H; **4** Me), 1.06 (s, 2.7 H; **2**(TMA) Me), 0.66 (t, 1.6 H; **4** CH_2_ *Me*), 0.62 (t, 1.4 H; **2**(TMA) CH_2_ *Me*), 0.10 (s, 1.4 H; **2**(TMA) Me_b_), −0.36 (s, br, 5 H; TMA), −0.47 (s, 5.5 H; **2**(TMA) Me_t_), −0.48 (s, 6.5 H; **4** Me_t_), −0.59 ppm (s, 2.8 H; **3** Me); ^13^C NMR (100 MHz, [D_6_]benzene): *δ*=79.5 (**2**(TMA) CO), 77.0 (**4** CO), 50.4 (**3** OMe), 48.3 (**4** OMe), 37.1 (**4** CH_2_), 36.6 (**2**(TMA) CH_2_), 28.1 (**4** Me), 27.7 (**2**(TMA) Me), 8.9 (**4** CH_2_ *Me*), 8.8 (**2**(TMA) CH_2_ *Me*), −4.5 (**2**(TMA) Me_b_), −7.5 (**4** Me_t_+**2**(TMA) Me_t_), −9.5 ppm (**3** Me).

### Thermal stability of 1:1 and 1:2 EtC(O)OMe 1:TMA reaction mixtures

The reaction mixture at the end of the 1:1 and 1:2 reactions of **1** with TMA in toluene (to give **1**(TMA), **2**(TMA) and **3**; see above) was heated to reflux for 2 hours using an oil bath. Aliquots (0.1 mL) were diluted with [D_6_]benzene (0.7 mL) and analyzed by NMR spectroscopy.

1:1 EtC(O)OMe **1**:TMA, *t=*2 hr. ^1^H NMR spectroscopy (400 MHz, [D_6_]benzene): *δ*=3.32 (s, 2.4 H; **1** OMe), 3.08 (s, 0.8 H; **3** OMe), 3.02 (s, 3 H; **4** OMe), 1.99 (q, 1.6 H; **1** CH_2_), 1.50 (q, 0.4 H; **2** CH_2_), 1.44 (q, 2 H; **4** CH_2_), 1.15 (s, 1 H; **2** Me), 1.10 (s, 6 H; **4** Me), 0.93 (t, 2.4 H; **1** Me), 0.69 (t, 0.5 H; **2** CH_2_ *Me*), 0.66 (t, 3 H; **4** CH_2_ *Me*), −0.42 (s, 1 H; **2** AlMe), −0.50 (s, 12 H; **4** Me_t_), −0.61 ppm (s, 1.6 H; **3** Me); ^13^C NMR (100 MHz, [D_6_]benzene): *δ*=173.4 (**1** CO), 77.3 (**2** CO), 77.0 (**4** CO), 50.5 (**1** OMe), 50.3 (**3** OMe), 48.3 (**4** OMe), 37.1 (**4** CH_2_), 37.0 (**2** CH_2_), 28.1 (**4** Me+**2** Me), 26.9 (**1** CH_2_), 9.0 (**2** CH_2_ *Me*), 8.8 (**4** CH_2_ *Me*+**1** CH_2_ *Me*), −9.5 ppm (br, **4** Me+**3**Me); ^27^Al NMR (104 MHz, [D_6_]benzene): *δ*=156.7 (**2**+**3**+**4**), 8.1 ppm (trace, unidentified).

1:2 EtC(O)OMe **1**:TMA, *t=*2 hr. ^1^H NMR spectroscopy (400 MHz, [D_6_]benzene): *δ*=3.06 (s, 0.5 H; **3** OMe), 3.01 (s, 3 H; **4** OMe), 1.50 (q, 0.3 H; **2** CH_2_), 1.43 (q, 2 H; **4** CH_2_), 1.15 (s, 0.9 H; **2** Me), 1.09 (s, 6 H; **4** Me), 0.68 (t, 0.4 H; **2** CH_2_ *Me*), 0.66 (t, 3 H; **4** CH_2_ *Me*), −0.40 (s, 0.9 H; **2** AlMe), −0.48 (s, 12 H; **4** Me_t_), −0.59 ppm (s, 0.9 H; **3** Me); ^13^C NMR (100 MHz, [D_6_]benzene): *δ*=77.3 (**2** CO), 77.0 (**4** CO), 50.4 (**3** OMe), 48.3 (**4** OMe), 37.1 (**4** CH_2_), 37.1 (**2** CH_2_), 28.1 (**4** Me+**2** Me), 9.0 (**2** CH_2_ *Me*), 8.9 (**4** CH_2_ *Me*), −6.1 (w, br, **2** Me), −9.5 ppm (br, **3** Me+**4** Me); ^27^Al NMR (104 MHz, [D_6_]benzene): *δ*=155.1 ppm (**2**+**3**+**4**).

### Spectroscopic characterization of BnC(O)OMe 5+TMA reaction mixtures

As for **1**+TMA but using methyl phenylacetate **5** (0.42 mL, 3 mmol) to give **5**(TMA), **6**(TMA) and **3**. An aliquot (0.1 mL) was mixed with [D_6_]benzene (0.7 mL) and analyzed by NMR spectroscopy.

1:1 **5**:TMA. ^1^H NMR spectroscopy (400 MHz, [D_6_]benzene): *δ*=7.13–6.86 (m, 5.5 H; **5**(TMA)+**6**(TMA) Ph), 3.34 (s, 2 H; **5**(TMA) CH_2_), 3.07 (s, 0.4 H; **3** OMe), 2.89 (s, 3 H; **5**(TMA) OMe), 2.86 (s, 0.2 H; **6**(TMA) CH_2_), 1.12 (s, 0.6 H; **6**(TMA) Me), −0.24 (m, 9 H; **1**(TMA), AlMe), −0.43 (s, 1.2 H; **6**(TMA) Me_t_), −0.59 ppm (s, 0.8 H; **3** Me); ^13^C NMR (100 MHz, [D_6_]benzene): *δ*=178.1 (**5**(TMA) CO), 136.6, 131.7, 130.3, 128.1, 127.6 (**5**(TMA+**6**(TMA) Ph), 79.5 (**6**(TMA) CO), 54.0 (**5**(TMA) OMe), 50.7 (**6**(TMA) CH_2_), 50.4 (**3** OMe), 40.7 (**5**(TMA) CH_2_), 28.1 (**6**(TMA) Me), −7.5 (**6**(TMA) AlMe), −9.3 (**5**(TMA) Me), −11.0 ppm (**3** Me); ^27^Al NMR (104 MHz, [D_6_]benzene): *δ*=186.3 (**5**(TMA)), 157.8 ppm (**3**+**6**(TMA)).

1:2 **5**:TMA. ^1^H NMR spectroscopy (400 MHz, [D_6_]benzene): *δ*=7.13–6.86 (m, 12.5 H; **5**(TMA)+**6**(TMA) Ph), 3.36 (s, 2 H; **5**(TMA) CH_2_), 3.09 (s, 4.5 H; **3** OMe), 2.89 (s, 3 H; **5**(TMA) OMe), 2.88 (s, 3 H; **6**(TMA) CH_2_), 1.13 (s, 9 H; **6**(TMA) Me), 0.14 (s, br, 4.5 H; **6**(TMA) Me_b_), −0.30 (m, 9 H; **5**(TMA), AlMe), −0.44 (s, br, 18 H; **6**(TMA) Me_t_), −0.61 ppm (s, 9 H; **3** Me); ^13^C NMR (100 MHz, [D_6_]benzene): *δ*=178.8 (**5**(TMA) CO), 136.6, 131.4, 130.3, 129.2, 128.6, 128.0, 126.7 (**5**(TMA+**6**(TMA) Ph), 79.5 (**6**(TMA) CO), 54.3 (**5**(TMA) OMe), 50.7 (**6**(TMA) CH_2_), 50.4 (**3** OMe), 40.7 (**5**(TMA) CH_2_), 28.0 (**6**(TMA) Me), −4.5 (**6**(TMA) Me_b_), −7.0 (**6**(TMA) Me_t_), −7.5 (**5**(TMA) Me), −11.2 ppm (**3** Me); ^27^Al NMR (104 MHz, [D_6_]benzene): *δ*=180.5 (sh, **5**(TMA)),153.6 ppm (**3**+**6**(TMA)).

1:3 **5**:TMA. ^1^H NMR spectroscopy (400 MHz, [D_6_]benzene): *δ*=7.15–6.84 (m, 5 H; **6**(TMA) Ph), 3.06 (s, 3 H; **3** OMe), 2.86 (s, 2 H; **6**(TMA) CH_2_), 1.11 (s, 6 H; **6**(TMA) Me), 0.13 (s, 3 H; **6**(TMA) Me_b_), −0.42 (s, 12 H; **6**(TMA) Me_t_), −0.59 ppm (s, 6 H; **3** Me); ^13^C NMR (100 MHz, [D_6_]benzene): *δ*=136.7, 130.3, 128.0, 126.7 (**6**(TMA) Ph), 79.5 (**6**(TMA) CO), 50.7 (**6**(TMA) CH_2_), 50.4 **(3** OMe), 28.0 (**6**(TMA) Me), −4.5 (**6**(TMA) Me_b_), −7.0 (**6**(TMA) Me_t_), −11.1 ppm (**3** Me); ^27^Al NMR (104 MHz, [D_6_]benzene): *δ*=154.0 ppm (**3**+**6**(TMA)).

### Co‐synthesis and characterization of BnMe_2_COAlMe_2_(TMA) 6(TMA) and 3

Method 1) TMA (4.5 mL, 9 mmol, 2.0 m in toluene) was added dropwise to methyl phenylacetate (0.42 mL, 3 mmol) under a N_2_ atmosphere at −78 °C and allowed to reach room temperature. The resulting solution was stirred and generated heat. After 2 hours the solution was placed under vacuum to remove the toluene. The remaining liquid was stored at 4 °C for 1 day, producing colourless crystals of **6**(TMA) and **3**. Combined yield of **6**(TMA) and **3**: 910 mg (83 % of the total mass expected); m.p. <30 °C; ^1^H NMR spectroscopy (400 MHz, [D_6_]benzene): *δ*=7.05‐6.84 (m, 5 H; **6**(TMA) Ph), 3.06 (s, 3 H; **3** OMe), 2.86 (s, 2 H; **6**(TMA) CH_2_), 1.11 (s, 6 H; **6**(TMA) Me), 0.13 (s, 3 H; **6**(TMA) Me_b_), −0.43 (s, 12 H; **6**(TMA) Me_t_), −0.60 ppm (s, 6 H; **3** Me); ^13^C NMR (100 MHz, [D_6_]benzene): *δ*=136.7, 130.3, 128.0, 126.7 (**6**(TMA) Ph), 79.5 (**6**(TMA) CO), 50.7 (**6**(TMA) CH_2_), 50.4 **(3** OMe), 28.0 (**6**(TMA) Me), −4.5 (**6**(TMA) Me_b_), −7.0 (**6**(TMA) Me_t_), −11.1 ppm (**3** Me); ^27^Al NMR (104 MHz, [D_6_]benzene): *δ*=153.8 ppm (**3**+**6**(TMA)); satisfactory elemental analysis could not be achieved; X‐ray crystal data: C_15_H_28_Al_2_O, *M*=278.33, orthorhombic, space group *Pbcm*, *a=*7.3370(4), *b=*17.3975(8) *c=*13.8976(6) Å, *V=*1773.97(15) Å^3^, *Z=*4, *ρ*_calcd_=1.042 g cm^−3^, *μ*=1.378 mm^−1^, *T=*180(2) K. 6976 data (1327 unique, *R_int_=*0.0377, θ<59.03 °) were collected. *wR*2={Σ[*w*(*F*_o_^2^‐*F*_c_^2^)^2^]/Σ[*w*(*F*_o_^2^)^2^]}^1/2^=0.1024, conventional *R=*0.0412 on *F* values of 1071 reflections with *F* ^2^ >2σ(*F* ^2^), *GoF*=1.072, 94 parameters. Max. peak/hole ±0.282 eÅ^−3^.

Method 2) As for Method 1 but after stirring the reaction mixture for 2 hours the solution was placed under vacuum to remove the toluene. The remaining liquid was treated with hexane (1 mL) and the resulting solution stored at −20 °C for 1 day, producing a small quantity of colourless crystals. Combined yield of **6**(TMA) and **3**: 240 mg (22 % of the total mass expected); m.p. 68–70 °C; ^1^H NMR spectroscopy (400 MHz, [D_6_]benzene): *δ*=7.05–6.84 (m, 5 H; **6**(TMA) Ph), 3.05 (s, 0.3 H; **3** OMe), 2.86 (s, 2 H; **6**(TMA) CH_2_), 1.11 (s, 6 H; **6**(TMA) Me), 0.12 (s, 3 H; **6**(TMA) Me_b_), −0.42 (s, 12 H; **6**(TMA) Me_t_), −0.58 ppm (s, 0.6 H; **3** Me); ^13^C NMR (100 MHz, [D_6_]benzene): *δ*=136.7, 130.3, 128.1, 126.7 (**6**(TMA) Ph), 79.5 (**6**(TMA) CO), 50.7 (**6**(TMA) CH_2_), 28.0 (**6**(TMA) Me), −4.5 (**6**(TMA) Me_b_), −7.0 ppm (**6**(TMA) Me_t_); ^27^Al NMR (104 MHz, [D_6_]benzene): *δ*=155.4 ppm (**3**+**6**(TMA)); satisfactory elemental analysis could not be achieved.

### Synthesis and characterization of BnMe_2_COAlMe_2_ 6

TMA (4.5 mL, 9 mmol, 2.0 m in toluene) was added dropwise to methyl phenylacetate (0.42 mL, 3 mmol) under a N_2_ atmosphere at −78 °C before being allowed to attain room temperature. The resulting solution was stirred and generated heat. After 2 hours the solution was placed under vacuum to remove the toluene. The remaining liquid was treated with Et_2_O (3 mL) to give a white precipitate that dissolved upon gentle heating. Colourless prismatic crystals formed as the mixture cooled to room temperature and over a period of 1 day produced a large crop of **6**. Yield 417 mg (67 % wrt TMA); m.p. 124–126 °C; ^1^H NMR spectroscopy (400 MHz, [D_6_]benzene): *δ*=7.07–6.92 (m, 5 H; Ph), 3.00 (s, 2 H; CH_2_), 1.22 (s, 6 H; Me), −0.28 ppm (s, 6 H; AlMe); ^13^C NMR (100 MHz, [D_6_]benzene): *δ*=137.1 (*i*‐Ph), 130.4 (*o*‐Ph), 128.3 (*m*‐Ph), 126.6 (*p*‐Ph), 77.6 (CO), 51.2 (CH_2_), 28.5 (Me), −5.7 ppm (AlMe); ^27^Al NMR (194 MHz, [D_6_]benzene): *δ*=179.8 ppm; elemental analysis calcd (%) for C_24_H_38_Al_2_O_2_: C 69.88, H 9.29; found: C 68.73, H 9.70; X‐ray crystal data: C_24_H_38_Al_2_O_2_, *M*=412.50, monoclinic, space group *P*2_1_/*n*, *a=*11.9568(5), *b=*8.7121(4), *c=*23.7485(10) Å, *β*=98.577(2) °, *V=*2446.19(18) Å^3^, *Z=*4, *ρ*_calcd_=1.120 g cm^−3^, *μ*=1.182 mm^−1^, *T=*180(2)K. 34306 data (4334 unique, *R_int_=*0.0301, θ<66.73 °) were collected. *wR*2={Σ[*w*(*F*_o_^2^‐*F*_c_^2^)^2^]/Σ[*w*(*F*_o_^2^)^2^]}^1/2^=0.0944, conventional *R=*0.0340 on *F* values of 4013 reflections with *F* ^2^ >2σ(*F* ^2^), *GoF*=1.063, 285 parameters. Max. peak/hole ±0.281 eÅ^−3^.

## Supporting information

As a service to our authors and readers, this journal provides supporting information supplied by the authors. Such materials are peer reviewed and may be re‐organized for online delivery, but are not copy‐edited or typeset. Technical support issues arising from supporting information (other than missing files) should be addressed to the authors.

Supplementary
