# Supplementary material for: Reactions of Trimethylaluminium: Modelling the Chemical Degradation of Synthetic Lubricants
Source: Chemistry. 2016 Dec 5;23(1):167–75. doi: 10.1002/chem.201604553 (PMC6680265; doi:10.1002/chem.201604553)
Supplement: Supplementary file 1 — Supplementary [file CHEM-23-167-s001.pdf]

CHEMISTRY
A European Journal

Supporting Information

Reactions of Trimethylaluminium: Modelling the Chemical Degradation of Synthetic Lubricants

Jonathan Slaughter, Andrew J. Peel, and Andrew E. H. Wheatley*[a]

chem_201604553_sm_miscellaneous_information.pdf

Front cover to be inserted here by typesetters

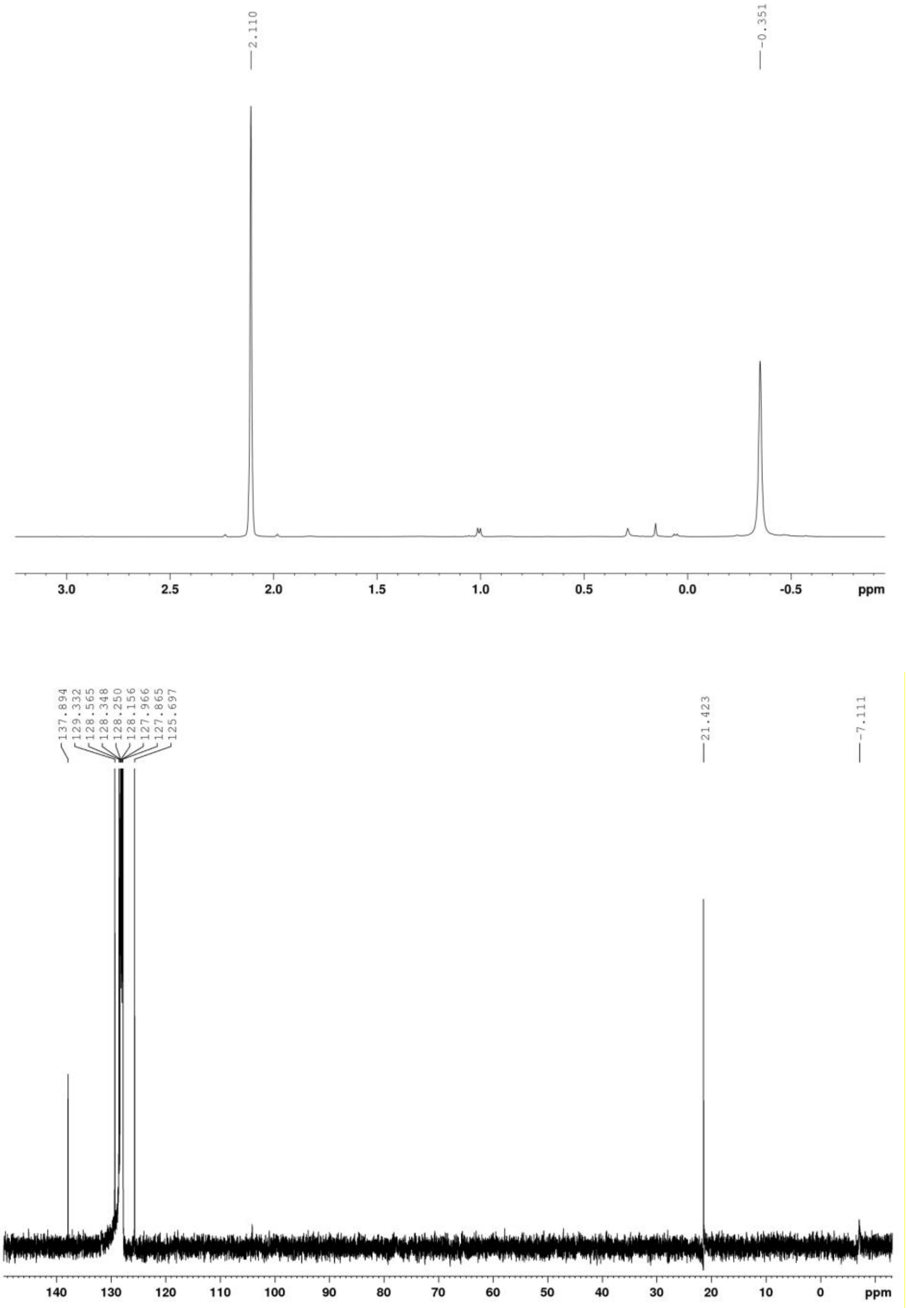

Figure S1 The ^{1}H (top) and ^{13}C NMR spectra (bottom) of TMA (2.0 M in toluene) in [D_6]benzene.

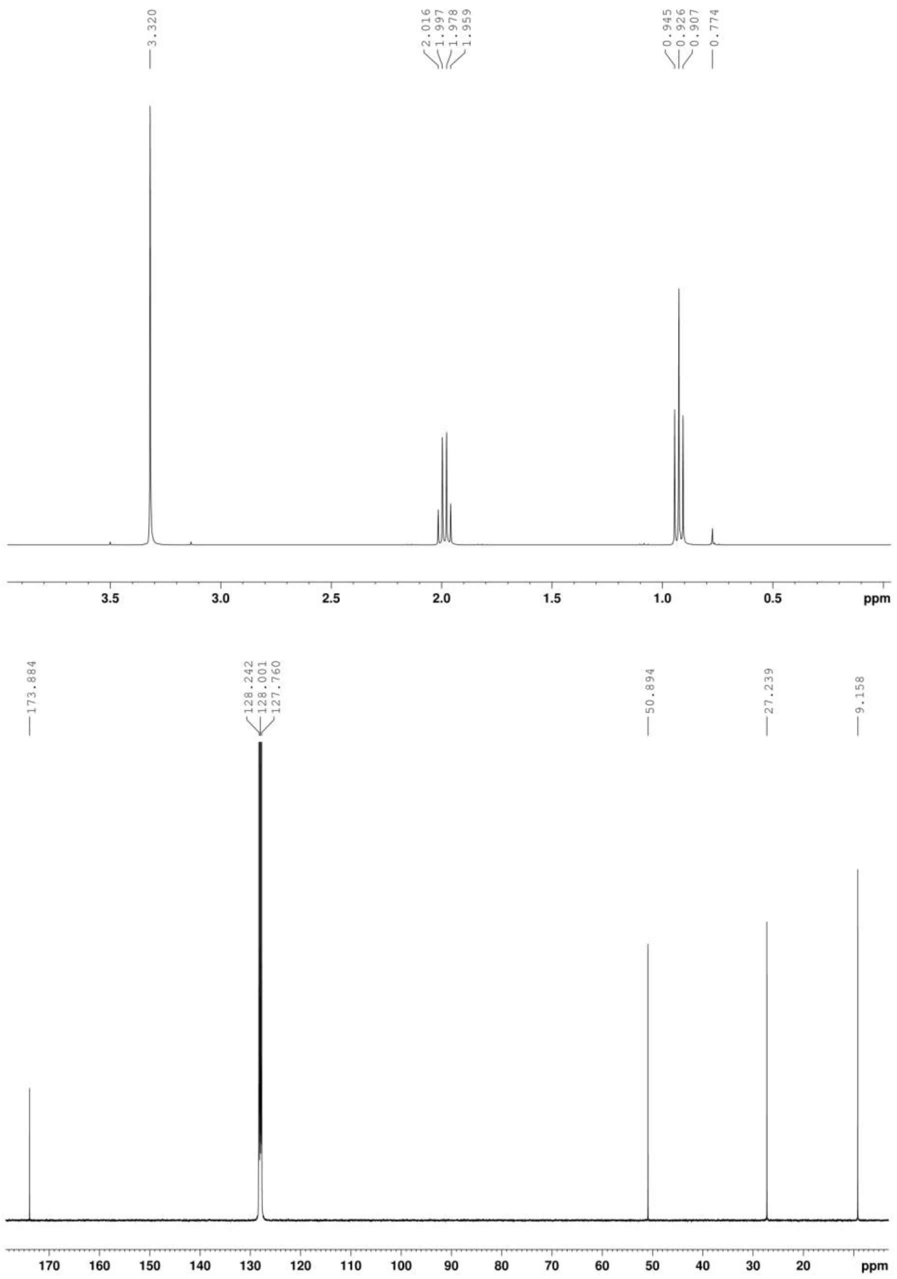

Figure S2 The ^{1}H (top) and ^{13}C NMR spectra (bottom) of methyl propionate **1** in [D$_6$]benzene.

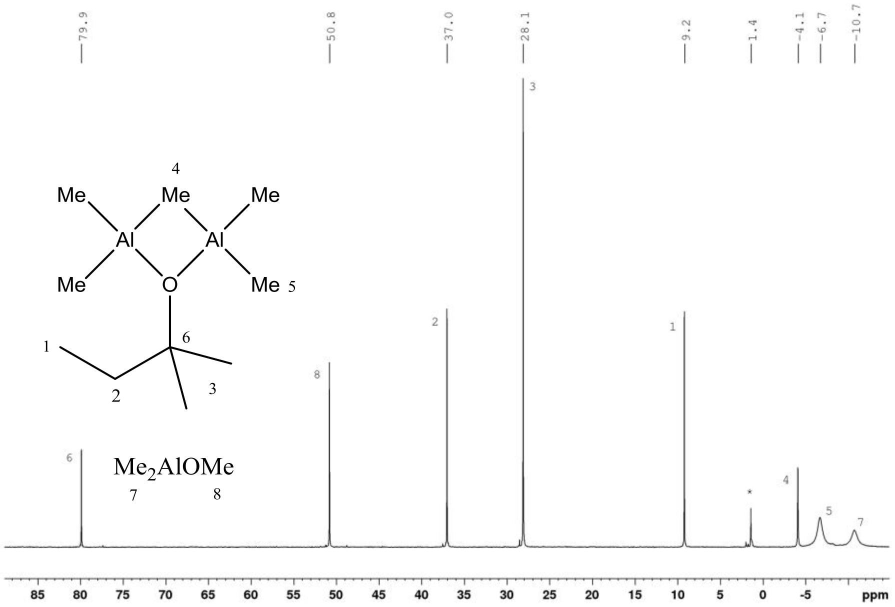

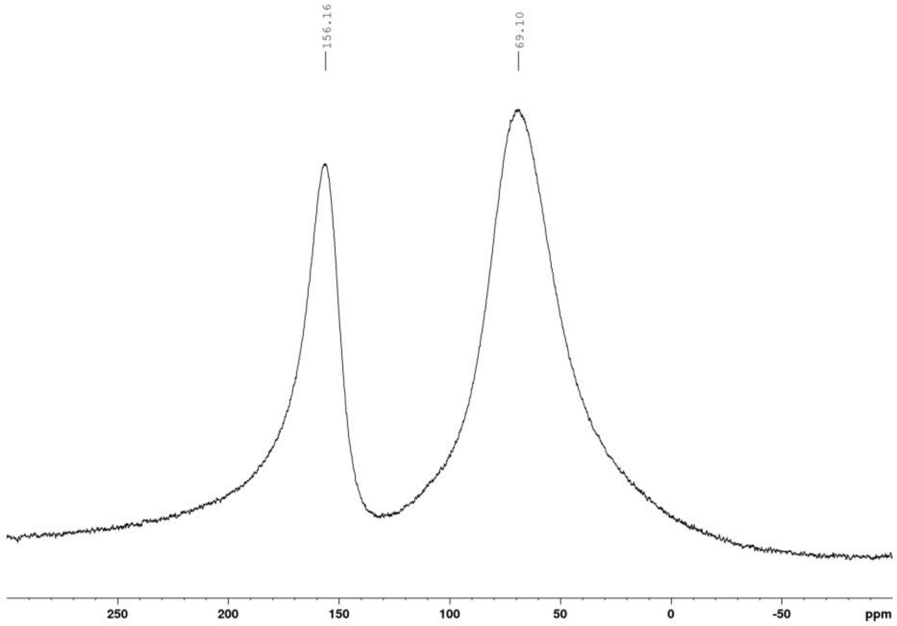

Figure S3 The ^{13}C (top) and ^{27}Al NMR spectra (bottom) of an aliquot from the reaction between a 3:1 mixture of TMA and **1** in toluene. The solvent is [D_6]benzene. *Silicon grease and the signal at δ 69.1 ppm in the lower spectrum is due to instrument components. The toluene has been removed from the products *in vacuo*.

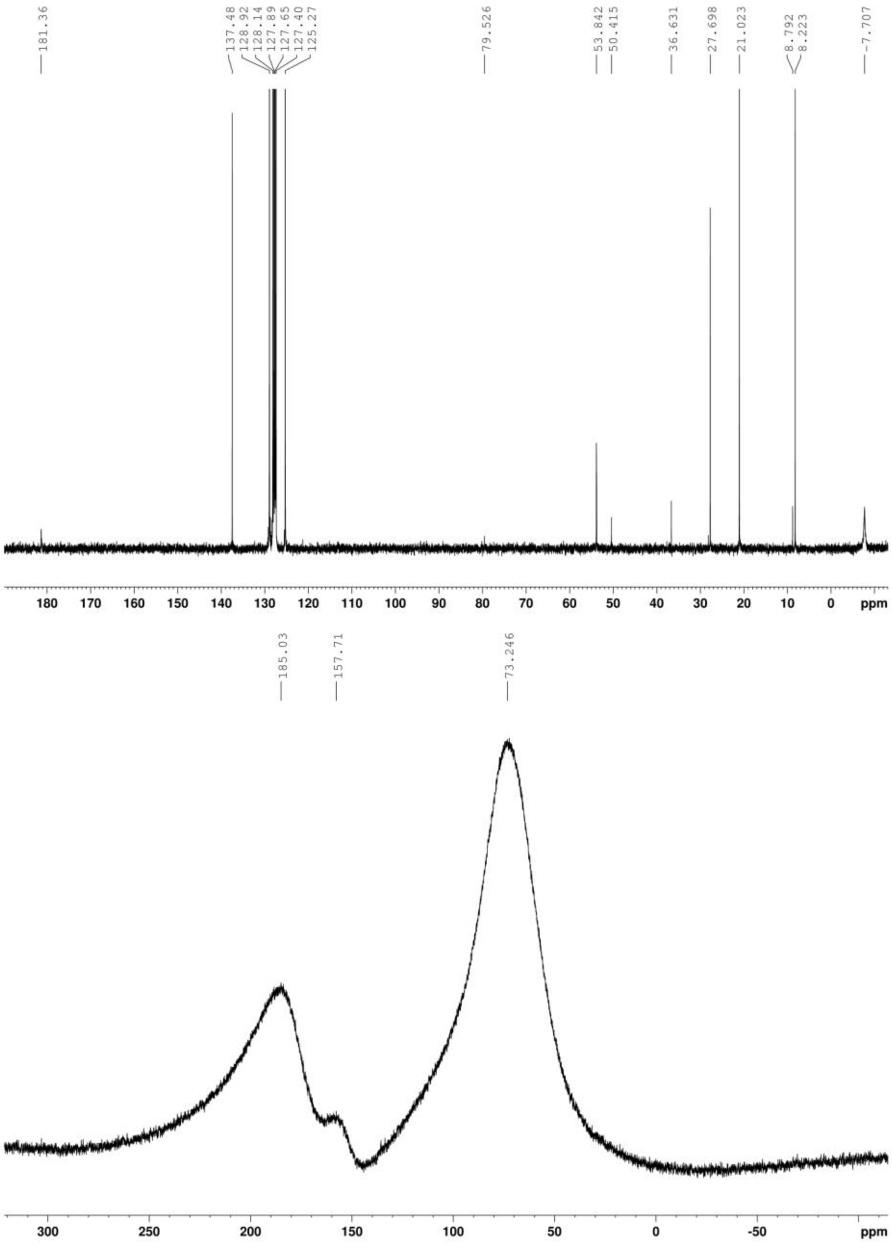

Figure S4 The ^{13}C (top) and ^{27}Al NMR spectra (bottom) of an aliquot from the reaction between a 1:1 mixture of TMA and **1** in toluene. The solvent is [D_6]benzene. The signal at δ 73.2 ppm in the lower spectrum is due to instrument components.

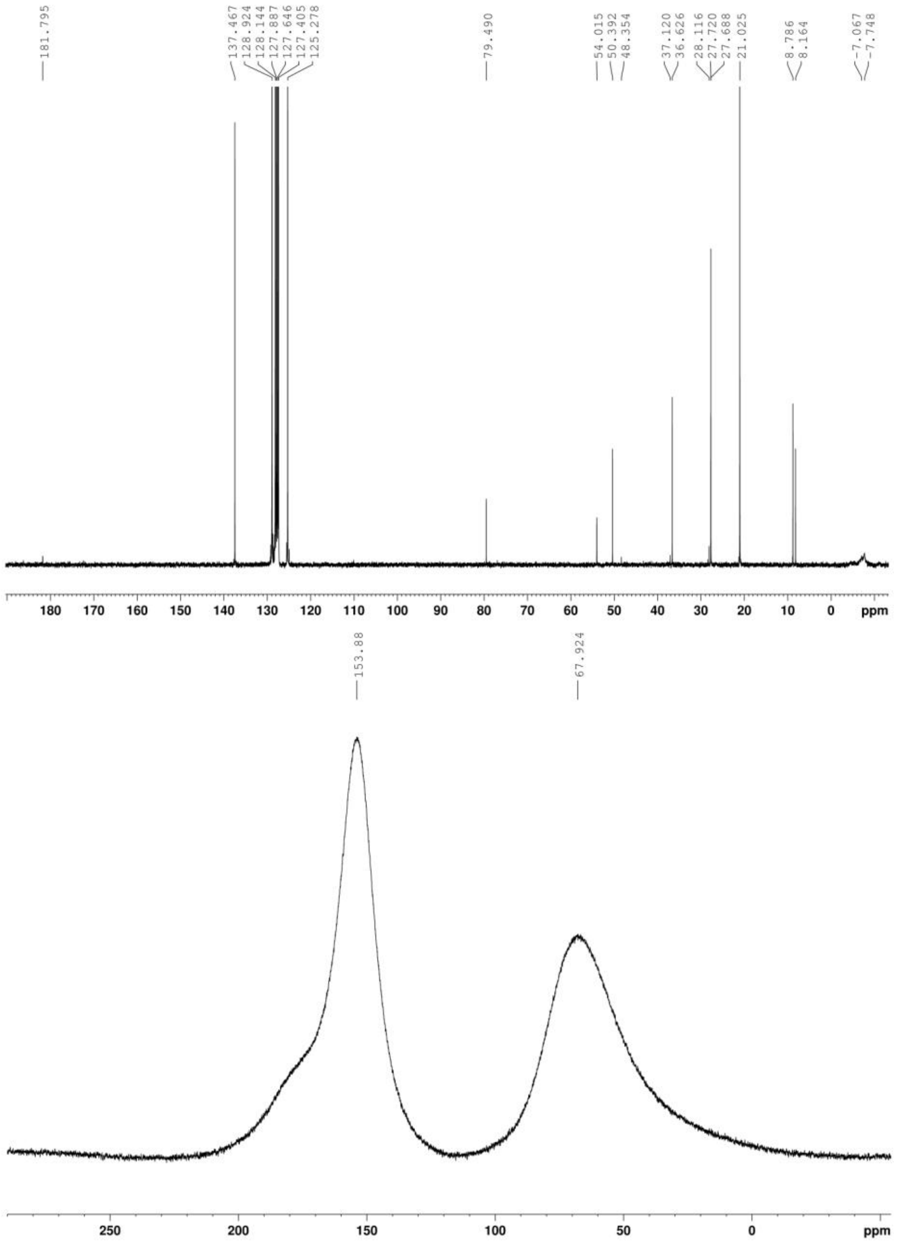

Figure S5 The ^{13}C (top) and ^{27}Al NMR spectra (bottom) of an aliquot from the reaction between a 2:1 mixture of TMA and **1** in toluene. The solvent is [D_6]benzene. The signal at δ 67.9 ppm in the lower spectrum is due to instrument components.

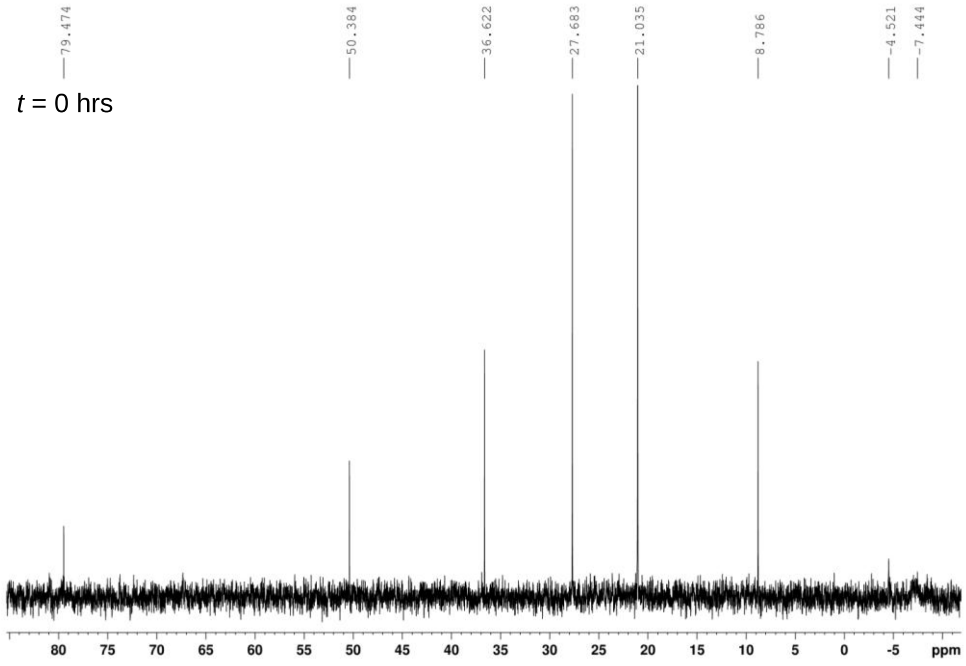

79.474
50.384
36.622
27.683
21.035
8.786
-4.521
-7.444
t = 0 hrs
80
75
70
65
60
55
50
45
40
35
30
25
20
15
10
5
0
-5
ppm

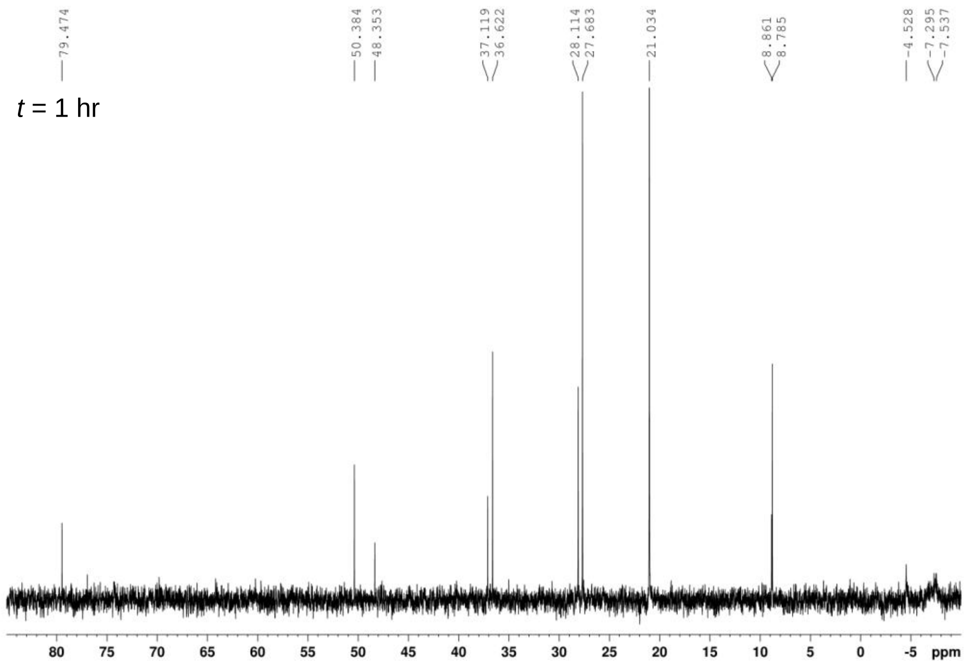

79.474
50.384
48.353
37.119
36.622
28.114
27.683
21.034
8.861
8.785
-4.528
-7.295
-7.537
t = 1 hr
80
75
70
65
60
55
50
45
40
35
30
25
20
15
10
5
0
-5
ppm

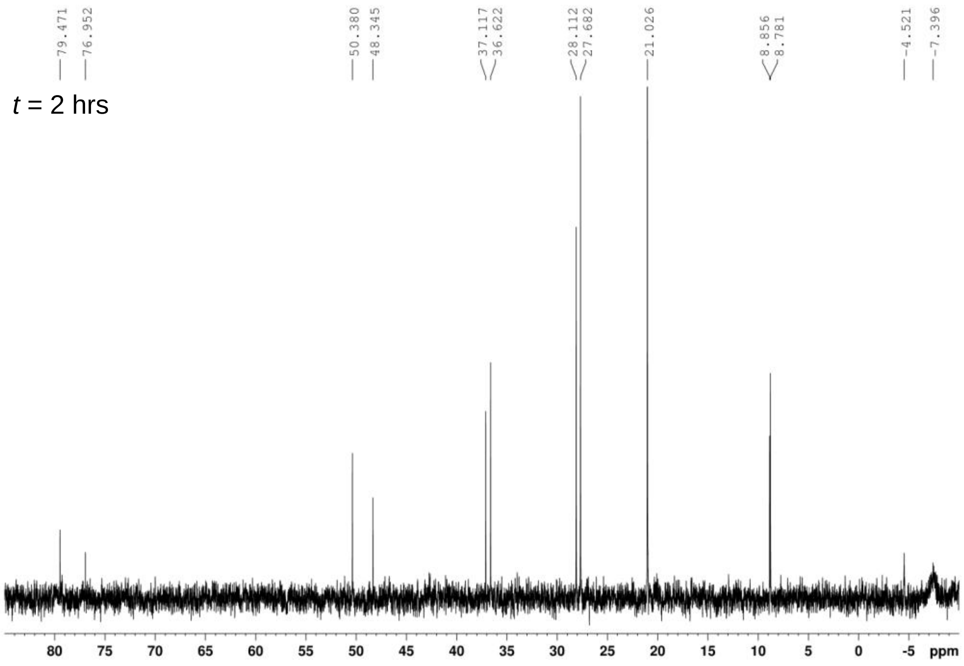
79.471
76.952
50.380
48.345
37.117
36.622
28.112
27.682
21.026
8.856
8.781
-4.521
-7.396
t = 2 hrs
80 75 70 65 60 55 50 45 40 35 30 25 20 15 10 5 0 -5 ppm

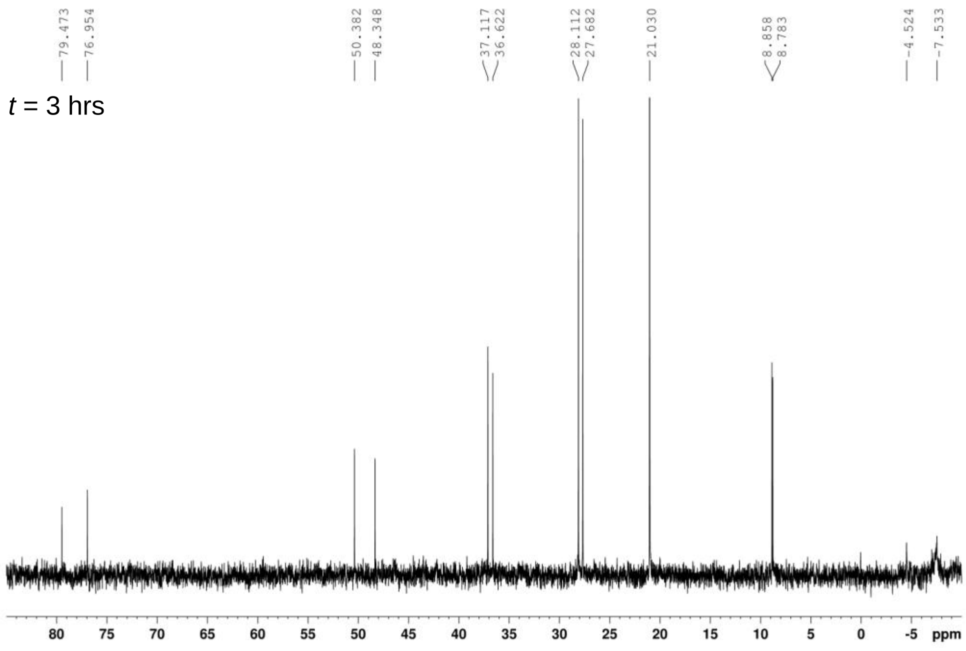
79.473
76.954
50.382
48.348
37.117
36.622
28.112
27.682
21.030
8.858
8.783
-4.524
-7.533
t = 3 hrs
80 75 70 65 60 55 50 45 40 35 30 25 20 15 10 5 0 -5 ppm

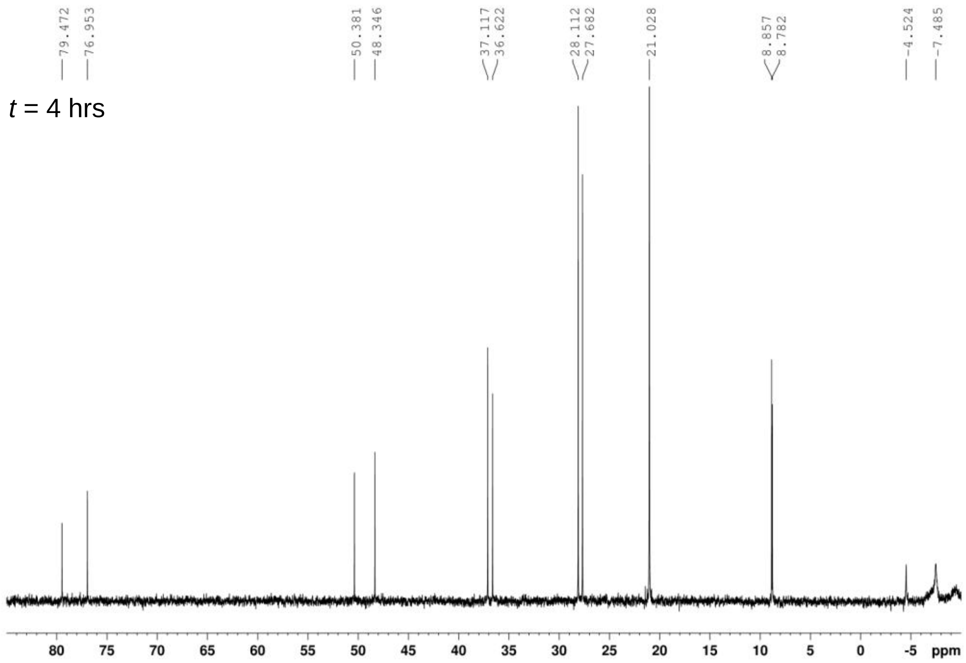

Figure S6 The ^{13}C spectra (locked using [D_6]benzene) of aliquots from the thermally induced (by heating to reflux) reaction between **2**(TMA) and **3** in toluene obtained after time (t) = 0-4 hours.

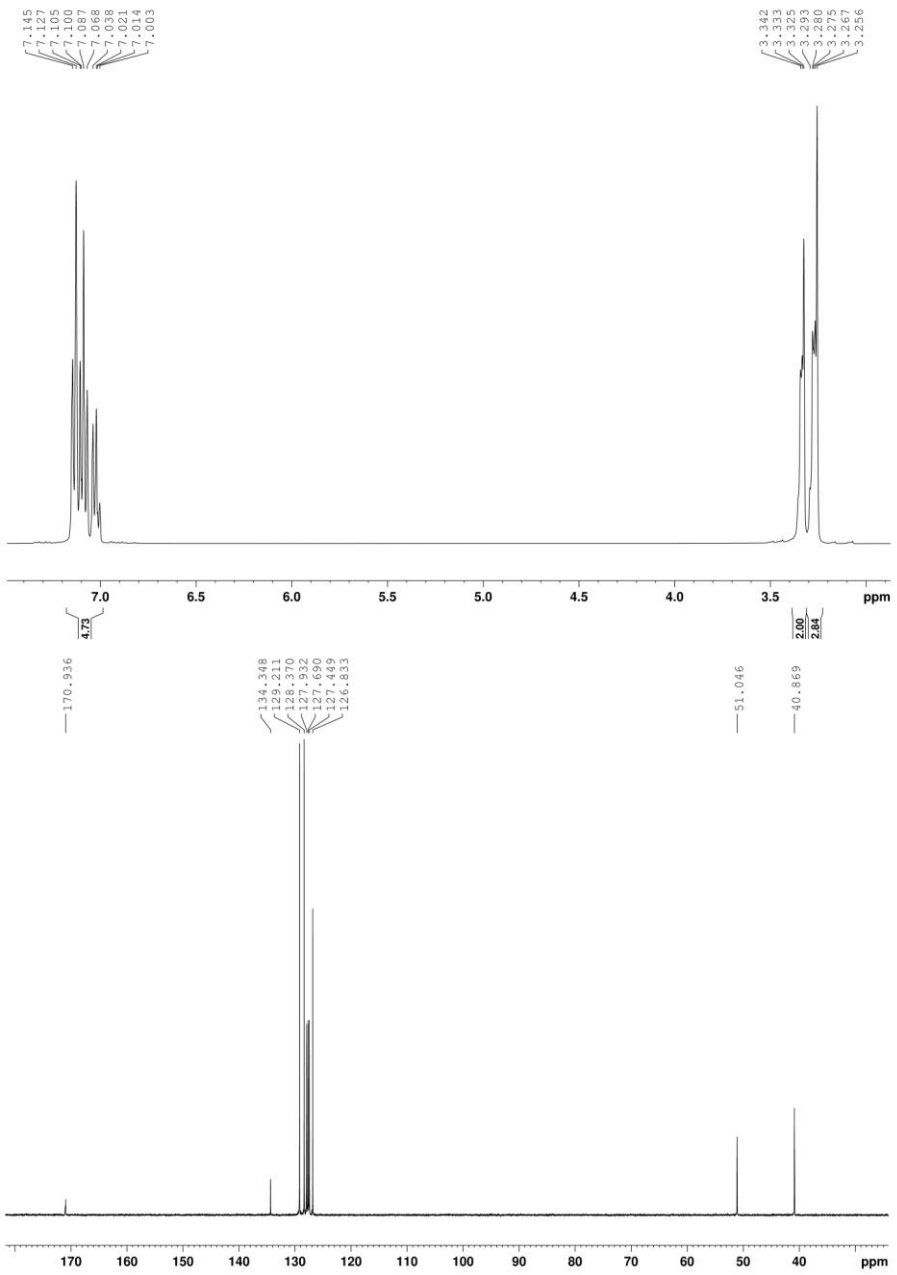

Figure S7 The ^{1}H (top) and ^{13}C NMR spectra (bottom) of methyl phenylacetate **5** in [D_6]benzene.

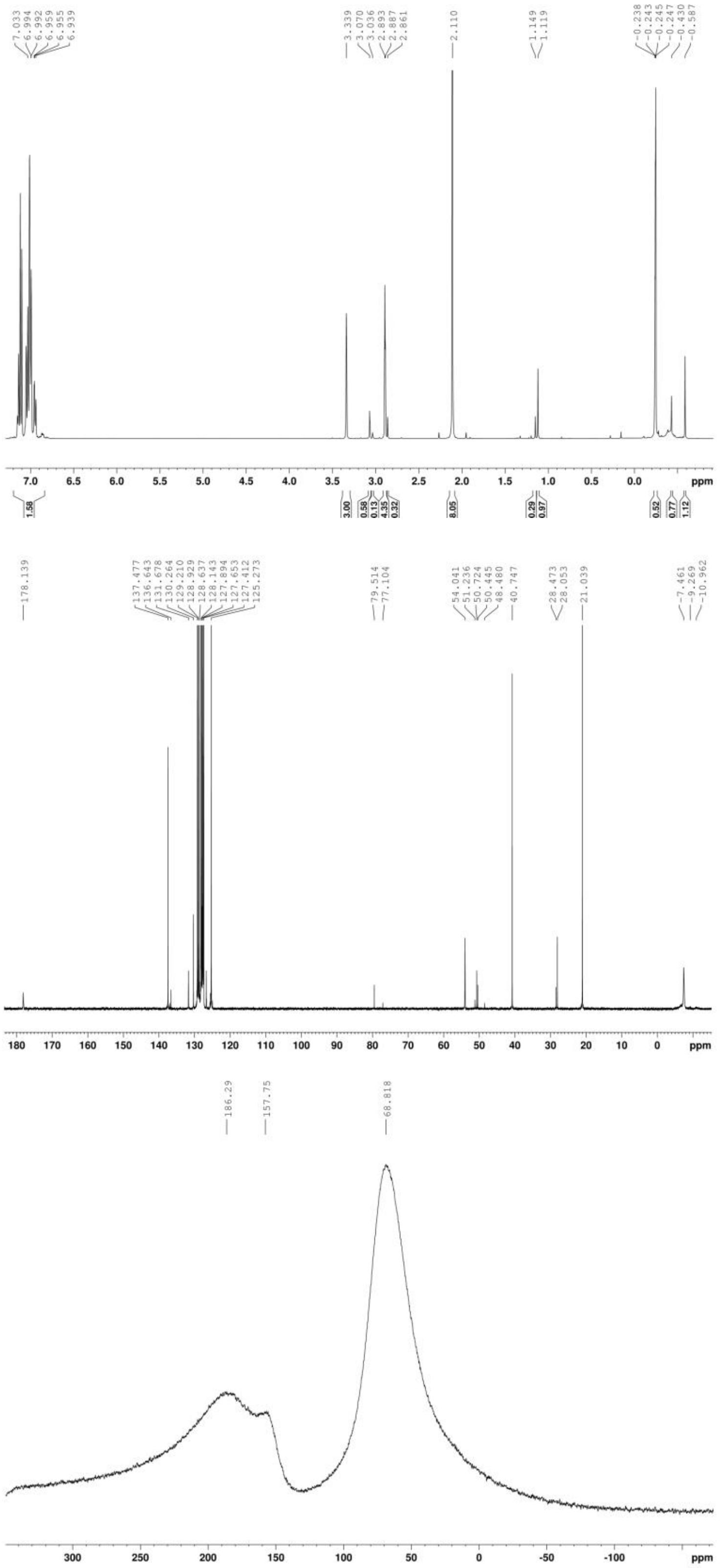

Figure S8 The ^{1}H (top), ^{13}C (middle) and ^{27}Al NMR spectra (bottom) of an aliquot from the reaction between a 1:1 mixture of TMA and methyl phenylacetate **5** in toluene. The solvent is [D$_6$]benzene. The signal at δ 73.2 ppm in the lower spectrum is due to instrument components.

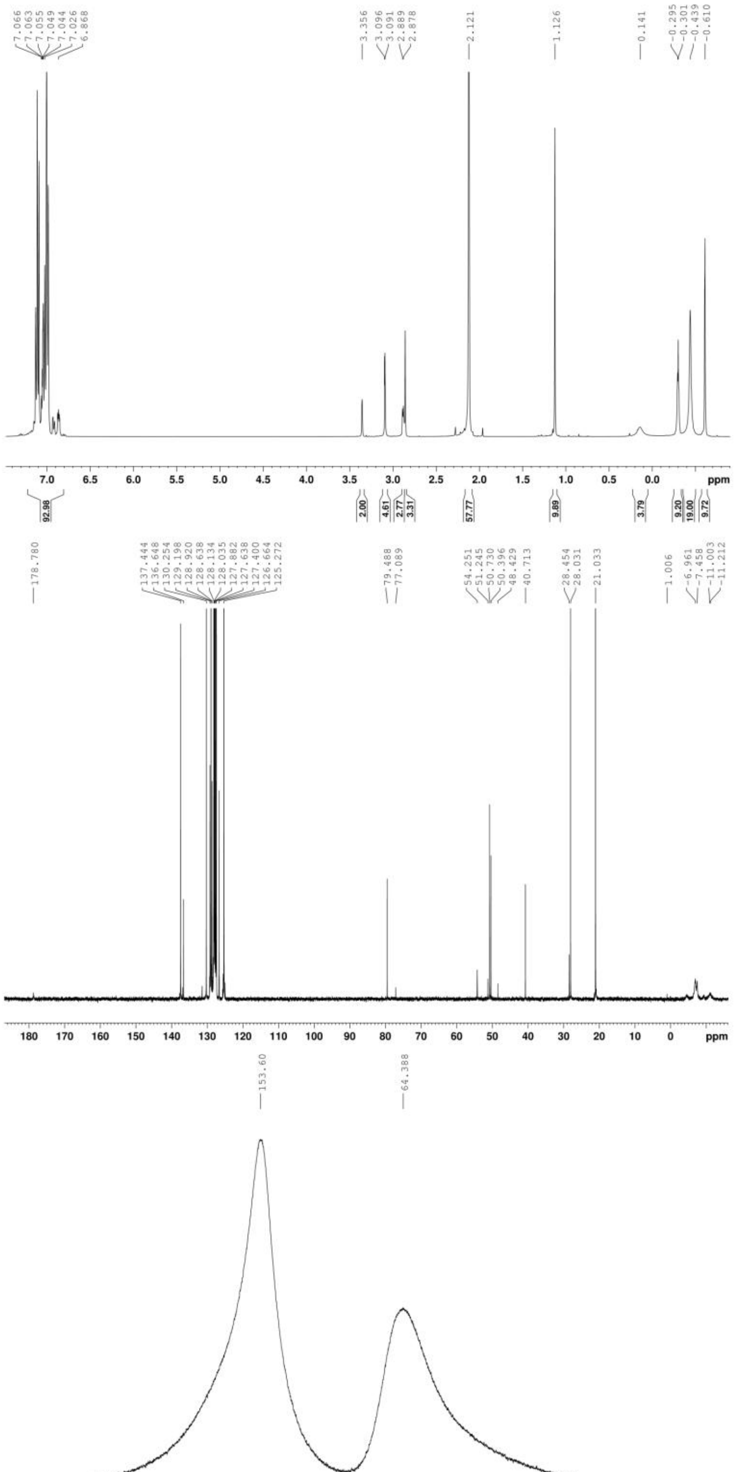

Figure S9 The ^{1}H (top), ^{13}C (middle) and ^{27}Al NMR spectra (bottom) of an aliquot from the reaction between a 2:1 mixture of TMA and **5** in toluene. The solvent is [D_6]benzene. The signal at δ 64.4 ppm in the lower spectrum is due to instrument components.

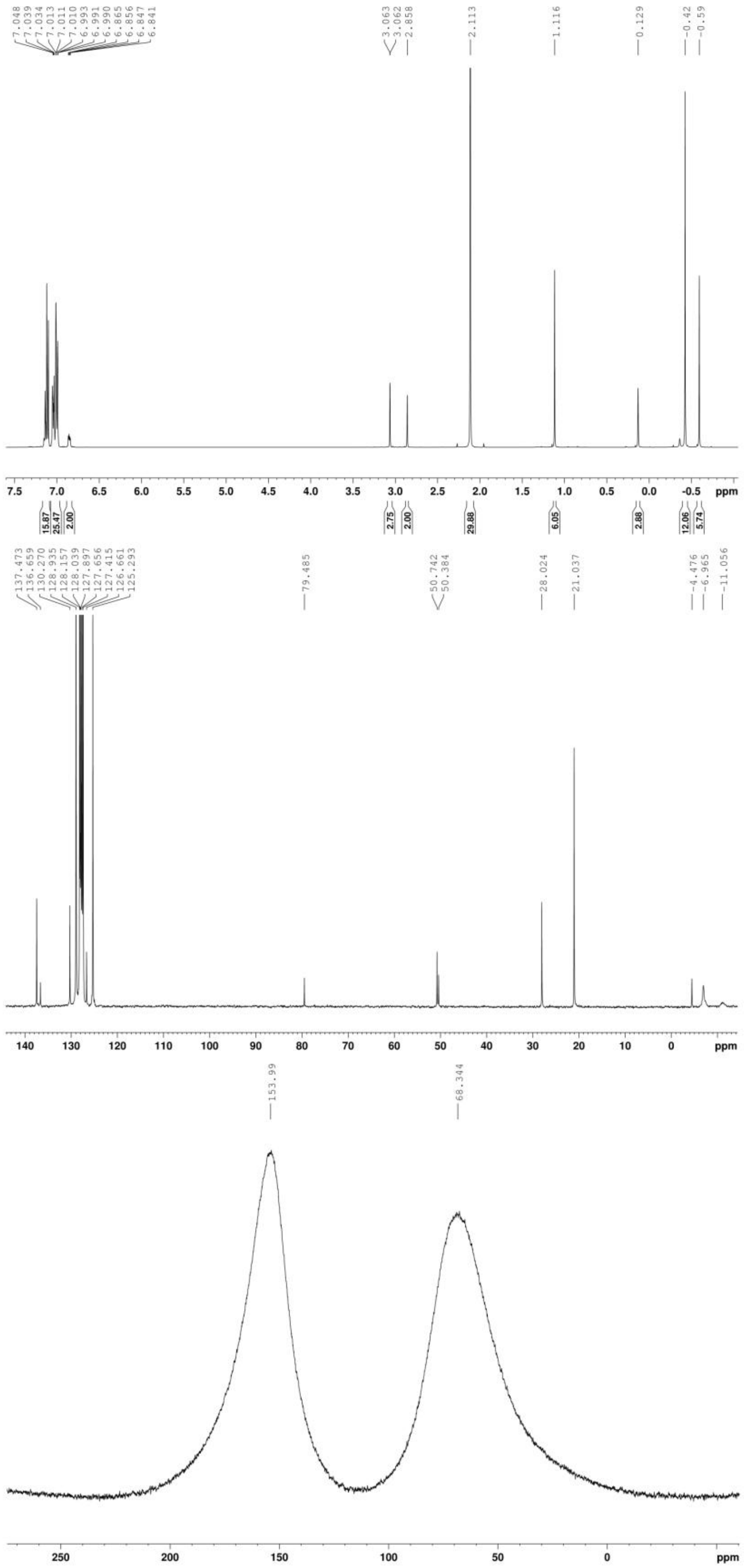

Figure S10 The ^{1}H (top), ^{13}C (middle) and ^{27}Al NMR spectra (bottom) of an aliquot from the reaction between a 3:1 mixture of TMA and **5** in toluene. The solvent is [D_6]benzene. The signal at δ 68.3 ppm in the lower spectrum is due to instrument components.
